# Molecular basis for anti-jumbo phage immunity by AVAST type 5

**DOI:** 10.1016/j.molcel.2026.01.004

**Published:** 2026-02-19

**Authors:** Aswin Muralidharan, Ana Rita Costa, Desi Fierlier, Daan Frits van den Berg, Halewijn van den Bossche, Adja Damba Zoumaro-Djayoon, Alicia Rodríguez-Molina, Martin Pabst, Martin Pacesa, Bruno E. Correia, Stan J.J. Brouns

**Affiliations:** 1 Department of Bionanoscience, Delft University of Technology, van der Maasweg 9, 2629 HZ Delft, the Netherlands; 2 Kavli Institute of Nanoscience, Delft University of Technology, van der Maasweg 9, 2629 HZ Delft, the Netherlands; 3 Department of Biotechnology, Delft University of Technology, 2629 HZ Delft, the Netherlands; 4 Laboratory of Protein Design and Immunoengineering, École Polytechnique Fédérale de Lausanne and Swiss Institute of Bioinformatics, Lausanne, Switzerland

**Keywords:** defense systems, *Pseudomonas aeruginosa*, jumbo phage, anti-jumbo phage immunity, AVAST, Sir2, STAND, TurboID, EPI vesicle

## Abstract

Jumbo phages protect their genomes from DNA-sensing bacterial defense systems by enclosing them within vesicles and nucleus-like compartments. Very little is known about defense systems specialized to counter these phages. Here, we show that AVAST type 5 (Avs5) systems, part of the signal transduction ATPases of numerous domains (STAND) superfamily, confer conserved immunity against jumbo phages. Using fluorescence microscopy and biotin proximity labeling, we demonstrate that Avs5 localizes to early infection vesicles, where it senses an essential, early-expressed phage protein named JADA (Jumbo phage Avs5 Defense Activator). Recognition of phage infection triggers the Sir2-like effector domain of Avs5 across three Avs5 clades, resulting in rapid NAD^+^ hydrolysis, disruption of phage nucleus formation, and arrest of infection. These findings reveal a spatially coordinated bacterial immune strategy that targets an early vulnerability in jumbo phage infection.

## Highlights

• Avs5 is activated at the early phage infection vesicle
• Avs5 hydrolyzes NAD^+^ to block jumbo phage nucleus formation
• Divergent Avs5 clades sense JADA to inhibit jumbo phage infection
• JADA is an essential early phage protein with a novel protein fold

## Graphical abstract

**Figure:**
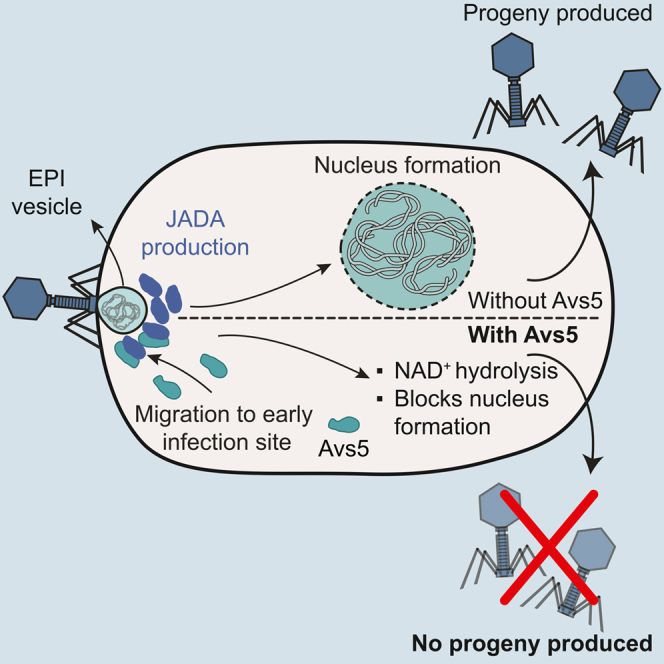

## Teaser

Muralidharan et al. reveal how the bacterial immune system AVAST type 5 (Avs5) blocks jumbo phage infection. Avs5 migrates to early phage entry sites and, upon binding to the essential viral protein JADA, triggers NAD^+^ depletion. This prevents the formation of the phage nucleus and blocks infection.

## Introduction

Bacteriophages of the *Chimalliviridae* family (also known as nucleus-forming jumbo phages) have evolved to possess an innate capability to elude detection by the prokaryotic immune systems like DNA-targeting CRISPR-Cas systems and restriction modification systems.^1^^,^^2^^,^^3^ To do so, they follow a carefully coordinated infection life cycle and sequential compartmentalization. During the early stages of infection, these phages compartmentalize their genome using an early phage infection (EPI) vesicle and evade detection by DNA-sensing defense systems.^4^^,^^5^^,^^6^^,^^7^ This evasion is achieved in the middle and late stages of infection through the physical concealment of their genome from bacterial cytoplasm within a selectively permeable proteinaceous nucleus-like genome replication and transcription compartment.^8^^,^^9^^,^^10^^,^^11^ This nucleus is enclosed by a self-assembling crystalline lattice (nuclear shell) composed primarily of the phage-encoded protein ChmA and is centered inside the bacterium by the phage-encoded tubulin-like filament PhuZ.^10^^,^^12^^,^^13^ The phage injects a multi-subunit virion RNA polymerase (vRNAP) along with the genome into the EPI vesicle to transcribe the early viral genes and a DNA polymerase, presumably for replication.^14^^,^^15^^,^^16^ To transcribe the late viral genes once in the nuclear shell, *Chimalliviridae* also carry a multi-subunit non-virion RNA polymerase (nvRNAP) composed of early gene products.^14^^,^^17^^,^^18^^,^^19^ The phage mRNA is transcribed inside the nucleus and is then translated outside the nucleus in the bacterial cytoplasm.^8^^,^^9^^,^^11^^,^^20^^,^^21^ The phage procapsids assemble outside the nucleus and then treadmill over PhuZ to dock onto the nuclear shell, where the genome is packaged into the capsid.^22^^,^^23^ The mature capsids then detach from the nuclear shell and accumulate with the assembled phage tail components to form mature phage particles.^8^ The host bacterium is then lysed, and the phage particles are released to infect new cells. Since the phage genome is concealed by the nucleus from the start of the infection till the end, the host must rely on defense systems that detect either phage mRNA or proteins to activate an immune response.

The strategies bacteria use to combat *Chimalliviridae* involve initiating immune responses through their adaptive and innate immune systems. While the phage DNA is protected in the nuclear shell from DNA-targeting CRISPR-Cas systems (types I, II, and VI), the RNA-targeting CRISPR-Cas systems (types III or VI) are effective by recognizing phage transcripts in the bacterial cytoplasm.^1^^,^^2^^,^^24^^,^^25^ Only a few innate defenses effective against *Chimalliviridae* have been identified,^26^ including Juk, the only characterized defense system specific to jumbo phages.^27^

Antiviral ATPases/NTPases of the STAND superfamily (AVAST) type 5, or Avs5, belongs to the ancient signal transduction ATPases of numerous domains (STAND) NTPase superfamily which plays a crucial role in pathogen-associated molecular pattern (PAMP)-triggered immunity across all domains of life.^28^^,^^29^^,^^30^^,^^31^^,^^32^^,^^33^^,^^34^^,^^35^^,^^36^ The STAND NTPases share a common tripartite modular domain architecture, featuring a central NTPase domain, a C-terminal sensor, and an N-terminal effector. STAND proteins in plants and animals are capable of dynamically transitioning between on and off states to regulate activity upon recognition of PAMPs, cytokines, and damage-associated molecular patterns.^37^^,^^38^^,^^39^^,^^40^^,^^41^ With evolutionary and structural similarities to universally used nucleotide-binding oligomerization domain (NOD)-like receptors (NLRs), Avs proteins represent a common link in the pattern recognition immune mechanisms between bacteria and other domains of life.^31^^,^^32^^,^^35^^,^^42^^,^^43^^,^^44^^,^^45^ The nuclease effector activity of Avs1-Avs4 leads to altruistic cell death by cleaving both the phage and host genomes. This occurs upon recognizing conserved structural patterns within the phage DNA packaging machinery, namely the large subunit of phage terminase and portal proteins or another phage protein, Ksap1, of unknown function.^29^^,^^35^ By direct recognition of these highly conserved and essential phage proteins, Avs systems provide host immunity against a broad range of phages.^29^^,^^33^^,^^36^^,^^46^ In contrast to the nuclease-based effectors of Avs1–4, Avs5 systems employ a Sir2 NADase. It remains an open question what activates Avs5 systems and what governs their phage specificity. Hence, we systematically studied the phylogeny, activation, and molecular mechanisms of Avs5.

We show that Avs5 systems form four distinct phylogenetic clades, of which three confer conserved immunity against nucleus-forming jumbo phage Pa36. One clade additionally mediates broad-spectrum defense. We show that the jumbo phage-specific *Pseudomonas aeruginosa* Avs5 (PaAvs5-1) system localizes to the EPI vesicle. Using proximity labeling, we identified that PaAvs5 from three clades recognizes an early-expressed, essential *Chimalliviridae*-specific protein, Pa36 gp316, here named JADA (Jumbo phage Avs5 Defense Activator). Cryo-EM analysis reveals that JADA forms a homodimer. Upon infection with a jumbo phage, these recognition events initiate nicotinamide adenine dinucleotide (NAD⁺) hydrolysis by the PaAvs5 Sir2 domain, disrupting a key metabolic cofactor. Using this metabolic disruption, PaAvs5 disrupts jumbo phage nucleus formation and slows progeny production. Together, our findings uncover the molecular basis, spatial localization, and temporal activation of Avs5 antiviral defense systems against jumbo phages.

## Results

### Shared anti-jumbo phage immunity by Avs5 homologs across phylogenetic clades

Recent studies showed that Avs5 systems, despite sharing a conserved domain architecture (Sir2-STAND), exhibit striking diversity in their anti-phage specificity across bacterial species.^30^^,^^36^ For example, Avs5 from *E. fergusonii* (EfAvs5) confers broad protection against diverse phages.^36^ By contrast, the *E. coli* Avs5 homolog (EcAvs5) exhibits a more targeted defense, providing immunity specifically against the T2 phage.^30^ Similarly, *P. aeruginosa* Avs5 (PaAvs5) specifically defends against the nucleus-forming jumbo phage Pa36.^26^ These contrasting immunity profiles, despite shared domain architecture, raise a key question: why do closely related Avs5 systems exhibit such divergent anti-phage specificities?

**Figure 1:**
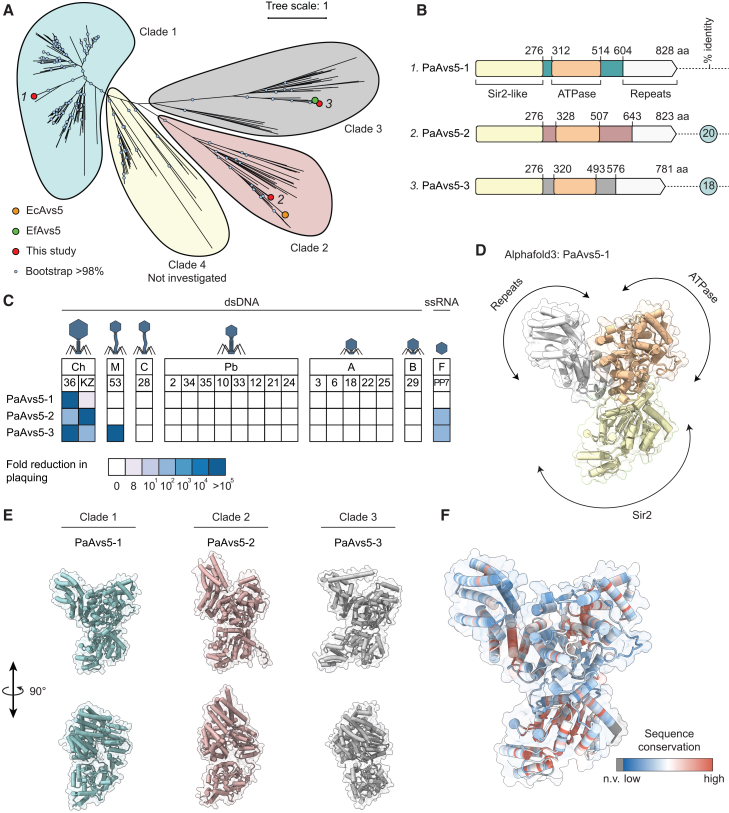
Phylogenetic distribution, domain architecture, and anti-phage immunity of Avs5 systems (A) Phylogenetic tree of Avs5 homologs, grouped into four major clades. The selected homologs are named with the clade number in superscript (i.e., PaAvs5-x is from clade x). The complete set of homologs used in this analysis is provided in Table S1. (B) Domain architectures of the three tested PaAvs5 systems. (C) Phage immunity profiles of PaAvs5 systems, shown as fold reduction in plaquing across a panel of phages from different families and genera: A, *Autographiviridae*; B, *Bruynoghevirus*; M, *Mesyanzhinovviridae*; F, *Fiersviridae*; Pb, *Pbunavirus*; Ch, *Chimalliviridae*; C, *Casadabanvirus*. (D) AlphaFold3 predicted structural model of PaAvs5-1. (E) AlphaFold3 predicted structural comparison of PaAvs5 homologs from each clade. (F) Sequence conservation mapped onto the PaAvs5-1 AlphaFold3 structure, based on a multiple sequence alignment of 334 PaAvs5 homologs. Residues without a conservation value (n.v.), including unaligned positions, are shown in gray.

To understand this divergence, we performed a phylogenetic classification of Avs5 homologs with a Sir2 effector domain. An unrooted phylogenetic tree revealed four distinct clades: PaAvs5 is found in clade 1 (PaAvs5-1), EcAvs5 in clade 2, and EfAvs5 in clade 3 (Figure 1A). To determine the range of phages against which Avs5 homologs confer immunity, we selected one representative homolog from each clade in our clinical *P. aeruginosa* collection (Figures 1A and 1B). These homologs were cloned into *P. aeruginosa* PAO1 using the low-copy pUCP20 plasmid under their native promoters. We then exposed these strains to a diverse panel of 19 phages from 7 families, including those from *Autographiviridae, Mesyanzhinovviridae*, *Bruynoghevirus*, *Casadabanvirus, Pbunavirus*, a single-stranded RNA (ssRNA) *Fiersviridae* phage, and two *Chimalliviridae* jumbo phages, all capable of infecting PAO1.^26^ PaAvs5-2 and PaAvs5-3 provided immunity against the ssRNA phage PP7, while PaAvs5-3 additionally protected against the *Mesyanzhinovviridae* phage Pa53 (Figure 1C). Notably, all tested Avs5 systems conferred strong immunity against the nucleus-forming jumbo phage Pa36, reducing its efficiency of plating by over five orders of magnitude (Figure 1C). Both PaAvs5-2 and PaAvs5-3 also provided robust protection against the jumbo phage ΦKZ (Figure 1C). By contrast, PaAvs5-1 did not fully suppress ΦKZ infection but produced smaller and fewer plaques, indicating partial restriction (Figure 1C). Among the tested systems, PaAvs5-1 displayed exclusive immunity to jumbo phages (Figure 1C).

Hence, our findings reveal striking plasticity in the anti-phage immunity profiles of Avs5 systems. In particular, the PaAvs5-3 conferred immunity against a remarkably diverse phage genera, including the ssRNA phage PP7, which carries only four coding sequences within a ∼3.6 kb genome; a siphophage with an ∼62 kb genome encoding 89 coding sequences; and a jumbo phage with a 280 kb genome encoding over 300 coding sequences. This variation mirrors previous observations on Avs5 systems in *E. coli* and *E. fergusonii*.^30^^,^^36^ While Avs5 homologs from different phylogenetic clades vary in the breadth of their phage targets, all retain conserved activity against nucleus-forming jumbo phages, revealing a shared core specificity.

### Structural and sequence variability in Avs5-sensing domains potentially drive immunity differences

To understand the molecular basis of Avs5 immunity, we checked its domain architecture (Figures 1B and 1D). Consistent with other Avs defense systems, the PaAvs5 we investigated shared a tripartite domain architecture. At the amino terminus, they are predicted to contain a silent information regulator 2 (Sir2)-like effector domain (Pfam: PF13289), nested within a DHS-like NAD/FAD-binding domain superfamily (SSF: SSF52467).^47^^,^^48^ Additionally, they are predicted to possess a novel STAND NTPase3 subdomain (nSTAND3, Pfam: PF20720).^28^^,^^49^ The carboxy-terminal repeat domains in the tested Avs5 systems are predicted to contain different types of structural repeats, including tetratricopeptide repeats (TPRs) and Sel1-like repeats in PaAvs5-1 and PaAvs5-2, and pentatricopeptide repeats (PPRs) in PaAvs5-3, which are often implicated in protein ligand binding.^50^^,^^51^

AlphaFold3 structural predictions suggest a potentially “closed” state, where the three domains adopt a compact configuration stabilized by extensive intradomain interactions (Figures 1D and 1E). In this predicted structure, the repeat domain appears to fold back onto the ATPase domain, possibly to regulate the oligomerization mediated by this domain (Figures 1D and 1E). Structural alignments of AlphaFold3 predictions revealed subtle variations among tested homologs, particularly in the organization of the sensing domain (Figure 1E). Pairwise structural alignments show similar folds, with PaAvs5-2 and PaAvs5-3 displaying TM-align scores of 0.68 and 0.67, respectively, in comparison to PaAvs5-1.

Pairwise alignments between tested homologs indicate low sequence identity across Avs5 homologs, with PaAvs5-1 and PaAvs5-2 sharing 20% identity (35% similarity), PaAvs5-1 and PaAvs5-3 sharing 18% identity (35% similarity), and PaAvs5-2 and PaAvs5-3 sharing 21% identity (35% similarity) (Figure 1B). Analysis of amino acid sequence conservation among homologs revealed poor conservation in the region between the Sir2 and nSTAND3 domains, the ATPase domain, and the carboxyl-terminal domain consisting of repeating alpha helices (Figures 1F and S1A–S1E).

Outside prokaryotes, the predicted structure of PaAvs5 shows strong similarity to Sterile alpha motif domain-containing protein 9/SAMD9 (Uniprot: Q5K651) from *Homo sapiens* (Foldseek^52^ BFMD database: probability = 1, E-value = 4.6 × 10^−16^, position in query = 3-818, TM score = 0.41, root-mean-square deviation [RMSD] = 13.6). Additionally, there is strong structural homology to the nucleotide binding adaptor shared by Apaf-1, R proteins, and Ced-4 (NB-ARC) domain-containing protein from *Cladophialophora carrionii* (Uniprot: A0A1C1CV94, Foldseek: probability = 1, E-value = 3.7 × 10^−6^, position in query = 199-825, TM score = 0.29, RMSD = 25.74). These proteins are implicated in innate immune responses, including antiviral roles.^53^^,^^54^ Despite low sequence identities (<15%), these structural homologies (Figure S1F) suggest shared cross-kingdom functional similarities.

Our results suggest that variability in both the sequence and structure of the carboxy-terminal repeat regions and the region between the Sir2 and nSTAND3 domains of the Avs5 homologs could play a role in recognizing distinct phage ligands. These structural differences may explain the diverse immunity profiles observed across the Avs5 clades. Given PaAvs5-1’s exclusive protection against the jumbo phage Pa36, we next sought to elucidate the molecular basis of its activation and immune response.

### PaAvs5 restricts phage propagation and delays jumbo phage propagation

**Figure 2:**
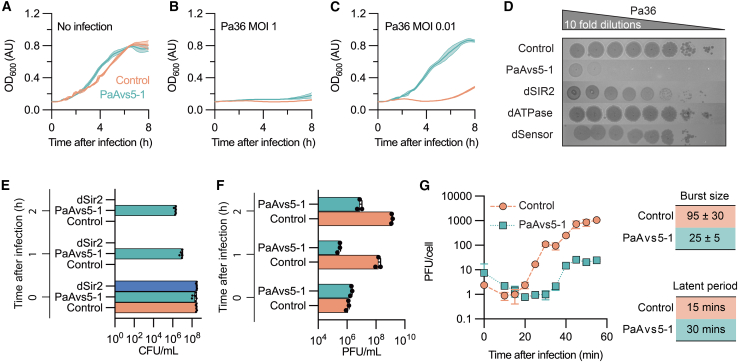
Jumbo phage resistance and infection kinetics of PaAvs5 (A–C) Growth curves of PAO1 strains expressing PaAvs5-1 or a control (empty plasmid) in liquid culture with or without Pa36 infection. (A) No infection control shows similar growth. (B) Infection with Pa36 at MOI = 1. (C) Infection with Pa36 at MOI = 0.01. (D) Efficiency of plaquing assay for Pa36 on wild-type PaAvs5-1 and inactivating mutants. (E) Bacterial survival measured in colony-forming units/mL (CFUs/mL) at different time points after infection by Pa36 (MOI = 10). (F) Phage replication measured in plaque-forming units/mL (PFUs/mL) at different time points after infection by Pa36 (MOI = 0.1). (G) One-step growth curve (MOI = 0.01) of Pa36 on control and PaAvs5-1-expressing strains, showing burst size and latent period.

To determine how PaAvs5-1 protects against Pa36, we infected strains with Pa36 at different multiplicities of infection (MOIs) in liquid culture. In the absence of infection, both control and PaAvs5-1 strains grew similarly (Figure 2A). At an MOI = 1, both strains underwent culture collapse (Figure 2B). At an MOI = 0.01, the PAO1 strain carrying PaAvs5-1 grew unaffected, while the control strain experienced culture collapse within 4 h (Figure 2C). Additionally, PaAvs5-1 strains reduced Pa36 plaquing efficiency by over five orders of magnitude compared with control strains (Figure 2D). Together, these results suggest that PaAvs5-1 may limit phage replication, possibly through altruistic cell death.

To assess whether PaAvs5-1 protects the host bacterium, we measured colony-forming units (CFUs/mL) after infecting PAO1 with Pa36 at an MOI = 10 for different durations (Figure 2E). PAO1 strains lacking functional PaAvs5-1 had no surviving colonies after infection for 1 h, indicating complete lysis. PAO1 strains carrying PaAvs5-1 showed only a ∼2-log reduction in CFU even after infection for 2 h, demonstrating that PaAvs5-1 provides protection under these conditions. In the high-MOI growth curve (Figure 2B), all cells are synchronously infected, leading to an immediate arrest in growth. By contrast, the single-round infection assay (Figure 2E) monitors surviving cells that recover and resume growth following successful activation of the defense system.

To assess whether PaAvs5-1 activity affects phage replication, we measured plaque-forming units (PFUs) after infecting the strains at MOI = 0.1 (Figure 2F). In control cells, phage replication increased from 10^6^ PFUs/mL before infection to nearly 10^9^ PFUs/mL within 2 h (Figure 2F). By contrast, PaAvs5-1-containing cells exhibited reduced phage titers during the first hour (Figure 2F). After 2 h, the titers in these cells showed only a modest increase, reaching 10^7^ PFUs/mL from an initial 2 × 10^6^ PFUs/mL (Figure 2F).

Next, we performed a one-step growth curve analysis to test the single replication cycle of the phage (Figure 2G). In the absence of PaAvs5-1, phage release (latent period) began approximately 15 min post-infection (mpi) (Figure 2G). However, in the presence of PaAvs5-1, the latent period doubled (Figure 2G). The burst size, or phage particles released per infected cell, was reduced from ∼95 PFUs/cell in control cells (and the second burst was ∼1,000 PFUs/cell) to ∼25 PFUs/cell in PaAvs5-1-expressing cells (Figure 2G). Combined, these effects resulted in an almost 100-fold reduction in total phage release 1-h post-infection (Figures 2F and 2G). Together with the strong population-level defense phenotype observed in plaque and growth assays, these results indicate that PaAvs5-1 restricts both single-cycle phage yield and subsequent propagation across the population.

Altogether, we demonstrate that PaAvs5-1 provides the host bacterium immunity by slowing jumbo phage propagation and reducing the efficiency of phage replication.

### Sir2 domain of PaAvs5 initiates NAD^+^ hydrolysis upon activation

**Figure 3:**
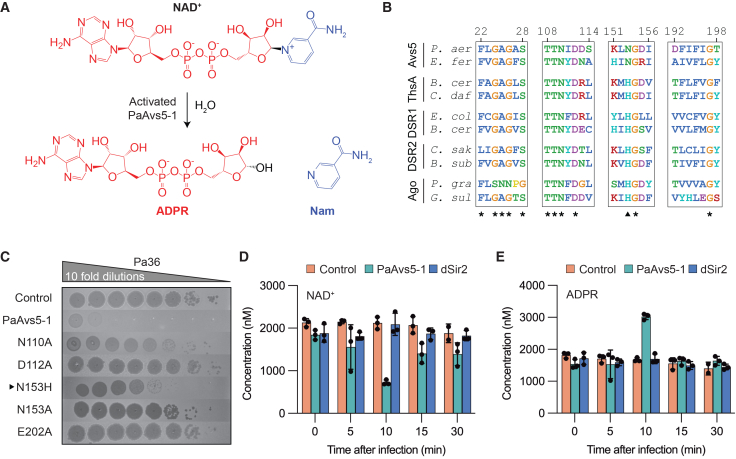
The Sir2 domain of PaAvs5 hydrolyses NAD^+^ upon activation (A) Schematic of NAD^+^ hydrolysis by activated PaAvs5-1, generating ADPR and Nam. (B) Multiple sequence alignment of the Sir2 domain across Avs5, ThsA, Ago, and DSR1/DSR2 homologs from diverse bacterial species. Conserved residues are marked (^∗^). Amino acids are colored based on the Clustal color scheme. (C) Efficiency of plaquing assay showing the impact of Sir2 domain mutations on PaAvs5-1-mediated defense against Pa36. (D and E) Intracellular NAD^+^ (D) and ADPR (E) concentrations measured in PAO1 cells infected with Pa36 (MOI = 3). PaAvs5-1 N110A mutant is represented as dSir2. Infection was initiated when cultures reached OD_600_ = 0.3. Data represent mean ± SD (*n* = 3 biological triplicates), with individual data points shown. Data are obtained using liquid chromatography-mass spectrometry (LC-MS).

The Sir2-like domain in PaAvs5-1 shares sequence similarity with the Sir2-like domains of ThsA from the Thoeris defense system and the DSR2 defense system, both of which hydrolyze NAD^+^ to trigger anti-phage activity.^55^^,^^56^ The proposed catalytic mechanism, like other Sirtuins, involves Asn110 coordinating a water molecule to position it near the ribose ring oxygen adjacent to the nicotinamide (Nam) ring of NAD^+^ (Figure 3A).^57^ Subsequently, this is expected to facilitate the hydrolysis of NAD^+^ to adenosine 5′-diphosphoribose (ADPR) and Nam upon activation.

Amino acid sequence alignments of the Sir2-like domains from Avs5, DSR1, DSR2, ThsA, and short prokaryotic Argonautes (Ago) reveal the presence of highly conserved motifs, GAGXS (a.a. 24–28 in PaAvs5-1) and TTNXD (a.a. 108–112), which are characteristic of all known Sirtuins (Figures 3B, S1B, and S1C). These motifs were previously shown to directly interact with NAD^+^ in Sirtuins.^58^ Site-directed point substitutions in the catalytic residues to alanine in the Sir2-like domain (N110A, D112A, and E202A) abrogated the immunity imparted by PaAvs5-1 (Figures 3C and S1G). This shows that the catalytic NAD^+^ hydrolysis activity of the Sir2 domain is essential for the PaAvs5-1 immune response.

Surprisingly, PaAvs5-1 lacks the canonical catalytic HG motif and instead harbors an NG (a.a. 153–154) motif in its place (Figures 3B and S1C). The HG motif, with the catalytic histidine, is considered to be absolutely conserved in Sir2 domains across domains of life.^58^^,^^59^^,^^60^ We found that the NG motif occurs nearly as frequently as the canonical HG motif among Avs5 homologs, appearing in approximately a one-to-one ratio (Figure S1C). Substituting Asn153 in PaAvs5-1 with histidine partially maintained activity but significantly reduced immunity (Figure 3C). Alanine or glutamine substitution of Asn153 abolished protection, indicating that Asn153 or His153 is required for defense, with Asn153 being more efficient (Figure 3C). The NG motif, therefore, is a novel variant of the canonical HG motif in the Sir2 domain.

To investigate the phage-activated NAD^+^ hydrolysis activity of the Sir2 domain in PaAvs5, we measured NAD^+^ and ADPR concentrations in Pa36-infected cells (MOI = 3, OD_600_ = 0.3). In PAO1 cells either lacking PaAvs5-1 or expressing a PaAvs5-1 variant with an inactive Sir2-like domain (N110A), NAD^+^ (∼2,100 nM) and ADPR (∼1,800 nM) levels remained stable during the first 30 min following Pa36 infection (Figures 3D and 3E). By contrast, cells expressing functional PaAvs5-1 exhibited a threefold decrease in NAD^+^ (∼700 nM) 10 mpi, accompanied by over a 50% increase in ADPR (∼3,000 nM) (Figures 3D and 3E). This demonstrates the successful hydrolysis of the nicotinamide-ADPR bond of NAD^+^ by the Sir2 domain to produce ADPR.

However, NAD^+^ concentrations in PaAvs5-1-expressing cells rebounded to basal levels within 15 mpi and remained stable through 30 mpi (Figure 3D). Similarly, ADPR concentrations returned to basal levels (∼1,600 nM) during the same period (Figure 3E). This suggests that ADPR may be recycled back into NAD^+^ by either jumbo phage-encoded NAD^+^-recycling proteins or host enzymes, but the exact cause is currently unknown. Nonetheless, the temporary reduction of the NAD^+^ pool appears sufficient for PaAvs5-1 to hinder Pa36 from successfully infecting the cell. Overall, our data suggest that the catalytic activity of the Sir2 domain is essential for the anti-phage defense of PaAvs5.

### ATPase activity of PaAvs5 is essential for immunity

The ATPase domain in PaAvs5-1 contains a phosphate-loop (P-loop) NTPase subdomain that often adopts a Rossmann fold and includes a Walker A motif (GPAGSGKT, amino acids 357–364, consensus Walker A motif: Gx_4_GK[S/T] with x representing any amino acids).^51^^,^^61^^,^^62^ This glycine-rich motif with an invariant lysine is commonly involved in the phosphate binding of ATP or GTP, with the Gly362, Lys363, and Thr364 residues in PaAvs5-1 coordinating the position of the triphosphate group (Figure S1B).^28^^,^^51^ Replacing the Gly362 or Lys363 with an alanine abrogated the PaAvs5-1 immunity (Figures 2D and S2). This indicates that NTP phosphate binding by the Walker A motif is essential for PaAvs5-1-mediated immunity.

Secondary structure predictions and AlphaFold3 modeling suggest that PaAvs5-1 contains a putative Walker B-like motif (IIFIE, residues 406–410). Walker B motifs are conserved elements in P-loop NTPases, typically consisting of four hydrophobic residues followed by two consecutive acidic residues.^61^ In PaAvs5-1, this motif has a single acidic residue (Glu410) instead of the canonical h_4_D[D/E] Walker B motif, where h represents any hydrophobic residue (Figure S1D). In PaAvs5-1, Arg411 is present within the ATPase pocket in place of a second acidic residue (Figure S1D). These residues are known to coordinate a catalytically essential Mg^2+^ ion for NTP hydrolysis.^28^^,^^51^ Mutating Glu410 to Ala abolishes the protective phenotype, while mutation of Glu410 to Gln retains the protective phenotype (Figure S1G). While the carboxylate group in Glu can coordinate Mg^2+^ directly and participate in proton abstraction to perform NTP hydrolysis, the amide group of Gln lacks these capabilities. Together, these findings suggest that the Walker B motif in PaAvs5-1 primarily facilitates NTP binding and conformational changes rather than directly catalyzing NTP hydrolysis. Additionally, Arg438, a highly conserved residue, is also essential for immunity. Its substitution to Ala partially eliminates protection against Pa36 (Figure S1F). Arg438 potentially serves a role as an arginine finger, often involved in forming contacts with the γ-phosphate of nucleotide.^28^

These findings indicate that NTP binding or hydrolysis by these residues is necessary for PaAvs5-1. Overall, our results show that the Walker A and Walker B motifs, along with the arginine finger, key elements involved in nucleotide binding, are required for PaAvs5 function.

### C-terminal repeat domain of PaAvs5 is essential for activation

To understand how PaAvs5 senses the PAMP, we examined its carboxy-terminal repeat domain, which likely serves a dual function: maintaining PaAvs5 in an inactive state while also acting as a pathogen sensor. Within this region, we identified two predicted repeat motifs in PaAvs5-1: one TPR motif (672–705) and one Sel1 motif (residues 713–748).^63^^,^^64^ AlphaFold3 structural predictions indicate that each motif adopts a tandem helix-turn-helix structure, with helices arranged in an anti-parallel fashion (Figure 1D). Repeat domains, such as these, are commonly involved in protein-protein interactions.^50^^,^^65^ Despite being poorly conserved, deletion of any predicted helices in the C-terminal region (PaAvs5-1 Δ812–828, PaAvs5-1 Δ795–828, or PaAvs5-1 Δ769–828) completely abolished PaAvs5-1-mediated immunity toward Pa36 (Figures 1C and S1G). Hence, this variable region near the C terminus plays a critical role in sensing phage infection and activating the defense response.

Toward the amino terminus of the repeat domain, we identified two highly conserved aromatic residues, Tyr661 and Trp675, and an invariant Gln677 (Figures S1B and S1E). Within the AlphaFold3 structural predictions, these residues cluster on consecutive anti-parallel α-helices within the repeat domain (Figure 1F). Its strict conservation and location bridging the TPR and ATPase domains suggest a role in serving as a pivot point for conformational switching.

### PaAvs5 inhibits formation of jumbo phage nucleus, and it migrates to the phage infection site

**Figure 4:**
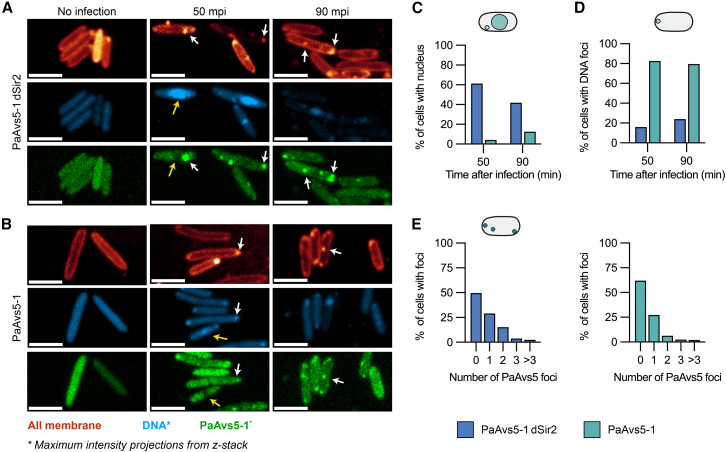
PaAvs5 inhibits formation of the jumbo phage nucleus and forms intracellular foci (A and B) Confocal microscopy images of jumbo phage-infected *P. aeruginosa* PAO1 strains expressing mNeonGreen-tagged (green channel) (A) PaAvs5-1 N110A and (B) PaAvs5-1. Representative PaAvs5-1 foci are indicated by white arrows. A yellow arrow highlights a phage nucleus and the corresponding region where PaAvs5-1 is excluded. DAPI and mNeonGreen-PaAvs5-1 channels are shown as maximum intensity projections from z-stacks, and the membrane channel is shown as a single optical section to highlight clear cell outlines. Contrast in the DAPI channel of uninfected cells was adjusted to improve visibility. Scale bar: 3 μm. (C) Quantification of the percentage of infected cells (from the same cells in C) showing visible phage nuclei at 50 and 90 mpi for strains expressing PaAvs5-1 N110A or wild-type PaAvs5-1. (D) Quantification of the percentage of infected cells showing visible DNA spots but no nucleus at 50 and 90 mpi for strains expressing either PaAvs5-1 dSir2/N110A (*n* = 131 cells at 50 mpi, 67 at 90 mpi) or wild-type PaAvs5-1 (*n* = 300 cells at 50 mpi, 137 at 90 mpi). (E) Quantification of the percentage of infected cells (from the same dataset shown in C) displaying visible PaAvs5 foci. Larger field of view microscopy images, along with additional data from dSensor and dATPase mutants, are provided in Figures S2A–S2D.

To study PaAvs5 localization and its effect on phage infection, we expressed a C-terminal mNeonGreen-tagged PaAvs5-1 from its native promoter in *P. aeruginosa* PAO1 using the pCUP20 plasmid. This fusion retained full protective activity against phage Pa36 (Figure S1G). Cells were infected at MOI = 5. Bacterial and phage DNA were visualized using DAPI staining, and cellular membranes, including phage-induced vesicles, were labeled with MitoTracker Deep Red-FM (Figures 4A and 4B).

At 50 mpi, imaging revealed clear differences between cells expressing wild-type and catalytically inactive PaAvs5-1. Approximately 60% of cells expressing the inactive PaAvs5-1 dSir2 mutant contained distinct phage nuclei (Figures 4A, 4C, and S2A–S2D). By contrast, fewer than 5% of cells expressing wild-type PaAvs5-1 formed a nucleus. Instead, ∼80% of these cells displayed localized DNA foci, typically near the poles (Figures 4B, 4D, and S2A–S2D), consistent with an immune response that arrests infection early. By 90 mpi, PaAvs5-1 dSir2 cells showed DNA degradation and lysis (Figure 4A), whereas wild-type PaAvs5-1 cells maintained intact genomes and rarely contained phage-induced nuclei (∼10%; Figures 4B and 4C).

In uninfected cells, PaAvs5-1-mNeonGreen was diffuse (Figures 4A, 4B, and S2A–S2D). Upon infection, the mNeonGreen signal was excluded from the phage nucleus and frequently concentrated into bright foci (∼40% of infected cells, *n* = 300), typically at the poles or along the membrane (Figures 4A, 4B, 4E, and S2A–S2D). Of these, ∼27% had a single focus, and ∼13% had multiple foci (Figure 4E). Similar patterns were observed for the catalytically inactive dSIR2 mutant (∼50% of cells, *n* = 131), indicating that focus formation does not require NADase activity (Figures 4A, 4E, and S2A). Focus intensity was ∼3–4× cytosolic background (when within dynamic range of imaging) and increased further in inactive mutants (Figures S2E–S2H). These foci were absent during Pa34 infection, a non-targeted phage, indicating functional specificity (Figure S3).

We then assessed whether these foci coincide with EPI vesicles. MitoTracker, previously used to visualize EPI vesicles in *E. coli* during Goslar jumbo phage infection,^6^ was less efficient in *P. aeruginosa*, so not all bacteria stained robustly (Figures 4A and 4B). However, in cells with adequate staining, MitoTracker-labeled foci colocalized with DAPI-labeled DNA foci and with PaAvs5-1-mNeonGreen foci (Figures 4A and 4B). This colocalization places PaAvs5-1 at membrane-associated compartments corresponding to EPI vesicles during early infection.

Recruitment to polar sites persisted in the sensor-deletion (Δ769–828) and ATPase-inactive (K363A) mutants (Figures S2B and S2C), suggesting that initial targeting is independent of the sensing or ATPase domains. Sequence analysis identified a predicted N-terminal hydrophobic localization segment (residues 2–30) within the Sir2-like domain.^66^^,^^67^ Deleting this region (Δ2–30 or Δ2–15) or substituting a non-conserved residue in this region (R9A) abolished protection (Figure S1G). Hence, N-terminal localization sequence appears critical for effective phage sensing and defense.

In summary, PaAvs5-1 mounts a spatially organized immune response by localizing to membrane-associated sites of phage entry. This spatial organization occurs independently of its catalytic or sensing functions. Upon infection, PaAvs5-1 accumulates at the poles and membrane, where it likely senses early phage proteins and blocks genome trafficking, preventing nucleus formation and halting infection.

### Identification of PaAvs5 activator via TurboID proximity labeling

**Figure 5:**
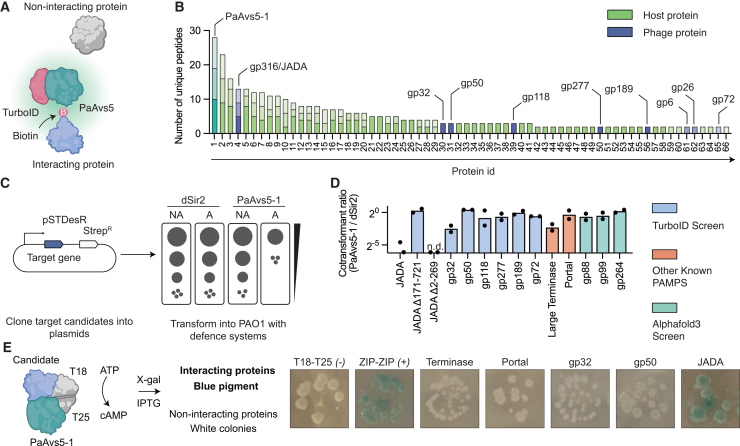
Identification and validation of phage proteins activating PaAvs5-1 (A) Schematic of the proximity biotinylation strategy using TurboID-tagged PaAvs5-1 to identify Pa36 phage proteins sensed by PaAvs5-1. Proteins in proximity to PaAvs5-1 are biotinylated by the TurboID tag, and non-interacting proteins remain unmodified. (B) Mass spectrometry analysis of biotinylated proteins showing the number of unique peptides identified per protein in PAO1 expressing PaAvs5-1-TurboID. Bars represent data from three biological replicates, with different transparency levels indicating individual replicates. gp316 is annotated as JADA. Details of protein ID are provided in Table S2. (C) Schematic of the co-transformation assay. Candidate genes were cloned under a rhamnose-inducible promoter (pSTDesR plasmid; streptomycin resistance) and transformed into PAO1 strains expressing either wild-type PaAvs5-1 or the dSir2/dSir2/N110A mutant (pUCP20 plasmid; carbenicillin resistance). (D) Transformant ratio (PaAvs5-1/dSir2) shown in log_2_ scale for each candidate gene. Data represent ratios of mean values from three independent transformations. n.d. indicates “not detectable,” corresponding to zero colonies observed in transformation assays. (E) Schematic of the BACTH assay used to test direct interactions between PaAvs5-1 and candidate phage proteins. Among the candidates tested, only the pair containing JADA and PaAvs5-1 produced blue colonies comparable to the ZIP-ZIP positive control, whereas other candidates yielded white colonies similar to the T18-T25 negative control, indicating no detectable interaction.

Based on the predicted protein-binding function of the TPR domain, we hypothesized that PaAvs5 is activated by a phage protein. To experimentally identify the phage protein responsible for activating PaAvs5-1’s enzymatic activity, we employed biotin proximity labeling.^68^ To do this, we fused TurboID, a promiscuous biotin ligase, to the N-terminal of PaAvs5-1 and cloned this fusion construct into PAO1. This fusion retained the phage defense activity of PaAvs5-1 (Figure S1G), and we hypothesized that phage proteins interacting with PaAvs5 would show enriched biotinylation (Figure 5A).

We found 66 unique biotinylated peptides from PAO1 cells carrying TurboID-PaAvs5-1 fusion when infected with Pa36 during our screening (Figure 5B; Table S2). The most abundant unique peptide was derived from PaAvs5-1 (Figure 5B; Table S2). Several abundant host proteins from PAO1, including phenylalanine-tRNA ligase alpha subunit, large ribosomal subunit proteins, and DNA-directed RNA polymerase subunits, show enriched biotinylation (Figure 5B; Table S2). Nine Pa36 proteins were biotinylated, of which only one protein, gp316, was enriched in all three biological replicates of the experiment (Figure 5B; Table S2). This was a strong indication that PaAvs5-1 interacts with gp316, a protein of unknown function, and could potentially be the protein sensed by the system to activate its Sir2 catalytic activity.

To test whether these proteins were the activator of PaAvs5-1, we introduced a low-copy pSTDesR plasmid encoding the phage proteins under a rhamnose-inducible promoter to PAO1 with and without the PaAvs5-1 system (Figure 5C). Our rationale was that PaAvs5-1 activation by the phage target protein would diminish transformation efficiency since co-expression of both PaAvs5-1 and the phage target protein would lead to NAD^+^ hydrolysis and growth arrest (Figure 5C). Only PAO1 cells expressing the dSir2 mutant, but not wild-type PaAvs5-1, were successfully transformed with gp316 (Figure 5D). This shows that gp316 activates the cell death function of PaAvs5-1. This verified that gp316 was one of the activators of PaAvs5-1 from our TurboID screen. Bacterial two-hybrid (BACTH) and pull-down assays further confirmed that PaAvs5-1 and gp316 directly interact (Figures 5E and S5). We then successfully cloned six out of the eight remaining Pa36 proteins that showed enriched biotinylation. All six were stably co-transformed with PaAvs5-1, and five of them showed similar transformation levels in both wild-type PaAvs5-1 and the dSir2 mutant (Figure 5D). gp32 showed nearly a twofold reduction in transformation efficiency, although the BACTH assay did not indicate a direct interaction with PaAvs5-1 (Figures 5D and 5E).

We then tested if the gp316 can be sensed by PaAvs5 systems from the other clades. Surprisingly, we observed that gp316 was able to activate both PaAvs5-2 and PaAvs5-3 despite its broader anti-phage immunity profile (Figure S6A). Given its ability to activate all tested homologs of the PaAvs5 defense system, we renamed Pa36 gp316 as JADA (accession number: XCN26233.1).

We further attempted to identify the activators of PaAvs5-1 using AlphaFold2 and AlphaFold3-based protein-protein co-folding. Out of 356 jumbo phage proteins screened, three candidates (gp88, gp99, and gp264) showed co-folding with PaAvs5-1, with ipTM scores above 0.7 and low predicted alignment error, suggesting potential interaction (Figure S4). However, none of these proteins activated PaAvs5-1 in experimental assays (Figure 5D).

We also tested whether homologs of known AVAST system activators, specifically the large terminase subunit and portal proteins, could activate PaAvs5 systems. The large terminase subunit caused a twofold reduction in transformants in wild-type PaAvs5-1 and PaAvs5-3 compared with the PaAvs5-1 dSir2 mutant (Figures 5D and S6A). Similarly, the portal protein induced a modest reduction in transformants in the PaAvs5-2 system compared with the dSir2 control (Figures 5D and S6A). However, none of these candidates triggered an immune response as strong as that induced by JADA, which was the only activator to elicit a robust response across all three Avs5 clades. BACTH assays further indicated no direct interaction between PaAvs5-1 and either the terminase or portal protein (Figure 5E). While we have not yet identified the activators from other phages, sequence homologs of gp316 were not found in the phages targeted by PaAvs5-2 and PaAvs5-3. This suggests that Avs5 systems can be triggered by multiple, distinct phage ligands. In summary, we identified an essential jumbo phage PAMP that can activate phylogenetically distant Avs5 systems.

### JADA is an early activator of PaAvs5

Since JADA activated all three PaAvs5 systems robustly, we next investigated its role in the jumbo phage lifecycle. Previous evidence suggests that JADA is essential for jumbo phage replication. Recent transcriptomics and proteomics data for phiKZ infection demonstrated that phiKZ_124, an ortholog of JADA, is essential, highly expressed, and found among the most abundant phage proteins ten mpi.^69^^,^^70^ The abundance and early expression of JADA are consistent with the rapid initiation of NADase activity of PaAvs5-1 upon Pa36 infection.

**Figure 6:**
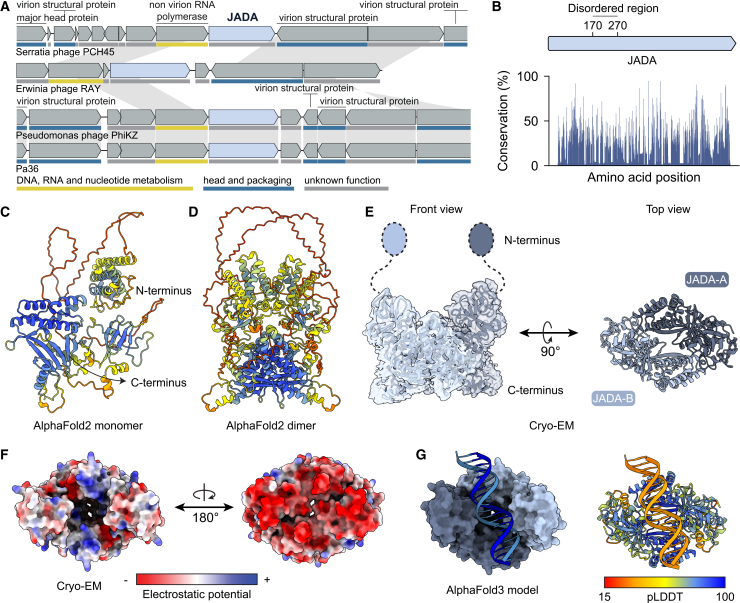
Structural characterization of JADA (A) Genomic context of JADA showing its conserved position immediately downstream of an nvRNAP subunit in different jumbo phages. Hypothetical genes are not labeled. Only the first instance of each homolog is annotated, with homologous genes across genomes connected by gray lines. Putative gene functions are indicated by the color scheme shown below. (B) Amino acid conservation across JADA, based on an alignment of 75 homologs from diverse phages. Sequence conservation is shown as the percentage occurrence of the most frequent amino acid at each position. (C) AlphaFold2-predicted monomeric structure of JADA colored by per-residue confidence (pLDDT; see G). (D) AlphaFold2-predicted dimeric structure of JADA, colored by per-residue confidence (pLDDT, see G). (E) Cryo-EM model of the JADA complex at 2.1 Å resolution (PDB ID: 9RP3; Table 1). (F) Electrostatic surface potential of the JADA from the cryo-EM structures. (G) AlphaFold3-predicted model of JADA bound to double-stranded DNA. Left: surface representation of the JADA-DNA complex. Right: ribbon view of the complex colored by pLDDT.

To investigate the role of JADA, we first analyzed its distribution among all phage genomes. This revealed that this gene was primarily present in phage genomes already classified as *Chimalliviridae* (Figure S6B). Even in distant jumbo phages, JADA is invariantly located directly downstream of a non-virion DNA-dependent RNA polymerase subunit (nvRNAP) and is transcribed in the same direction, suggesting coordinated expression (Figures 6A and S6C). Few conserved residues were identified in the JADA protein (Figure S6D).

We attempted to characterize the interaction between PaAvs5-1 and JADA, but the PaAvs5-1 protein proved too unstable for *in vitro* biochemical assays or cryo-EM studies. Hence, we focused on understanding the structure and potential role of JADA. AlphaFold2 structural predictions (pTM = 0.53) show that JADA comprises an N-terminal domain (residues 1–170) connected to a C-terminal domain (residues 270–721) by a long disordered or flexible region (Figures 6B–6D). High-resolution cryo-EM (2.1 Å) and mass photometry of recombinantly purified JADA expressed in *E. coli* BL21-AI revealed a rigid C-terminal dimer (Figures 6E and S7A–S7F; Table S3). The density map shows no evidence of bound ligands or ions within the central cavity. The base of the dimer is characterized by a highly negatively charged surface, a feature that may underlie function (Figure 6F). Additionally, no density was observed for the N-terminal region of the protein. Only the region corresponding to the C-terminal domain was solved by cryo-EM, consistent with the predicted disordered linker.

Fluorescence microscopy further revealed that JADA localizes to EPI vesicles. When expressed as an mScarlet3 fusion in cells co-expressing PaAvs5-1 dSir2-mNeonGreen, JADA displayed diffuse cytosolic fluorescence prior to infection (Figure S7G). Upon infection with phage Pa36, distinct JADA foci emerged near concentrated DAPI-stained DNA regions corresponding to EPI vesicles colocalizing with PaAvs5-1 dSir2 foci (Figure S7H). These findings suggest that JADA is associated with the EPI vesicle during early stages of infection.

The N-terminal domain was well predicted in isolation (pLDDT > 90) and appears structurally stable but lacks significant Foldseek matches above a TM score of 0.5, aside from hits to hypothetical proteins from related *Pseudomonas* phages in the Big Fantastic Virus Database (BFVD).^52^^,^^71^ AlphaFold2 and AlphaFold3 models indicate that the N-terminal domain does not form homodimers (Figure 6D).

The C-terminal domain of JADA also lacks significant structural homologs, with Foldseek multimer failing to identify any confident hits in the PDB or BFMD databases (closest hit TM score ∼0.3). The closest structural match for the full-length JADA monomer in the AlphaFold database has a TM score of 0.57, again corresponding to a related *Pseudomonas* hypothetical phage protein. Functional annotation of JADA via ProteInfer suggests a potential DNA-binding and regulatory role.^72^ Consistent with this function, AlphaFold3 modeling of the JADA C-terminal allows plausible DNA docking within a positively charged surface cavity of the homodimer (Figure 6G).

To determine which region of JADA activates the PaAvs5 system, we generated domain deletion constructs within the same pSTDesR plasmid: one encoding only the N-terminal domain (JADA Δ171–721) and another encoding only the C-terminal domain (JADA Δ2–269). When expressed alone, the N-terminal domain failed to activate PaAvs5 (Figure 5D). By contrast, co-expression of the C-terminal domain with inactive PaAvs5 yielded very few or no transformants under standard induction conditions, suggesting that the C-terminal domain may be toxic even in the absence of PaAvs5 activity. We then repeated the transformation assay under non-induced conditions, relying on leaky plasmid expression. Under these conditions, we recovered transformants with the inactive PaAvs5 system but not with the active PaAvs5 from all three clades (Figure 5D). These results indicate that the C-terminal domain of JADA is sufficient to activate Avs5 systems from all three clades.

## Discussion

**Figure 7:**
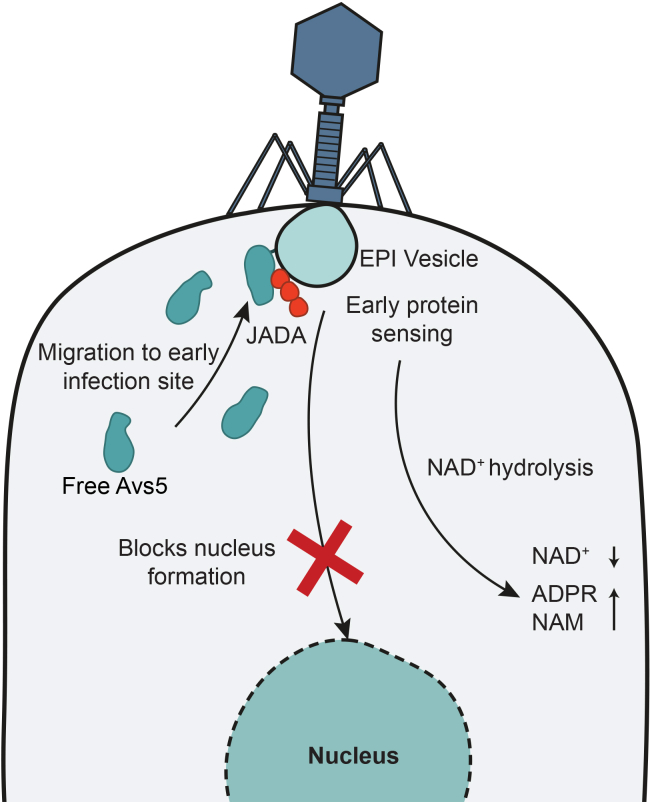
Model for Avs5-mediated defense against jumbo phages Following infection, Avs5 migrates to the early infection site, where it senses the early-expressed phage protein JADA. This sensing activates NAD^+^ hydrolysis by Avs5, leading to a transient hydrolysis of cellular NAD^+^.

Among the various strategies in the ongoing bacteria-phage arms race, the sequential genome compartmentalization employed by *Chimalliviridae* phages (nucleus-forming jumbo phages) is particularly unique. This highly regulated lifecycle through sequential compartmentalization of phage genome allows them to evade all known DNA-targeting defense systems.^1^^,^^2^^,^^4^^,^^6^ In response, bacteria have evolved alternative, specialized strategies that bypass DNA targeting altogether, instead recognizing phage-derived transcripts or proteins to trigger defense responses.^25^^,^^26^^,^^27^ Here, we demonstrate that PaAvs5Avs5 systems spanning three distinct clades display a conserved, robust defense against nucleus-forming jumbo phages (Figure 7). The nucleus-forming ability of jumbo phages is severely impacted by PaAvs5. In most cells containing PaAvs5, jumbo phage infection is halted at the EPI vesicle stage. Such a strong response might be mediated by rapid sensing and migration of PaAvs5 to infection sites. PaAvs5 forms multiple distinct intracellular foci that colocalize with the EPI vesicle. This suggests PaAvs5 is recruited to this early transcription site, where polysomes emerge from the vesicle surface. These foci are not coupled to sensing alone, as inactive sensor mutants, Sir2 mutants, and ATPase mutants were able to form such foci.

Upon sensing, PaAvs5-1 triggers a rapid drop in NAD^+^ levels due to strong hydrolysis within 10 min of infection, consistent with activation by an early-stage phage factor. NAD^⁺^ and its hydrolysis product ADPR return to near-basal levels within the next 10 min. While the mechanism remains unclear, the phage may encode NAD^+^ recycling enzymes that convert ADPR back to NAD^+^.^73^^,^^74^ Despite the transient nature of this response, it is sufficient to protect the host. NAD^+^ is an essential cofactor for both metabolism and gene expression.^75^ The rapid NAD^+^ hydrolysis triggered by PaAvs5 activation likely collapses cellular energy metabolism. Our results indicate that this depletion coincides with the earliest, non-recoverable phase of phage transcription. This likely prevents synthesis of phage proteins required for nucleus formation, thereby blocking successful assembly. Disruption during this narrow “window of opportunity” irreversibly aborts the infection, even if NAD^+^ levels subsequently recover. While several studies have linked Sir2-mediated NAD^+^ hydrolysis to phage defense,^75^^,^^76^ the precise molecular mechanism by which this activity confers protection remains an open question.

PaAvs5 systems, though exhibiting diverse anti-phage activity profiles and low sequence similarity, converge on sensing the same early-expressed Pa36 jumbo phage protein to initiate an immune response. We identified and validated this shared trigger, an essential protein we named JADA. Remarkably, JADA activates PaAvs5 systems across all three phylogenetic clades, enabling a robust defense that suppresses phage plaques by over five orders of magnitude. High-resolution cryo-EM of purified JADA homodimer revealed a novel protein fold with no identifiable sequence or structural homologs outside of related jumbo phages. JADA is composed of two structural domains, with the larger C-terminal domain responsible for PaAvs5 activation. Furthermore, its conserved genomic positioning next to the nvRNAP and gene ontology predictions point to a possible role in transcription during EPI. Together with the observed colocalization of PaAvs5 and JADA with the EPI vesicle, these findings suggest that JADA functions in early phage gene expression, a process that PaAvs5 likely suppresses to block infection.

The ability of PaAvs5-3 to defend against three unrelated phages, two jumbo phages, an RNA phage, and a siphophage indicates that its activation is not restricted to a single viral protein but may instead involve multiple triggers. These findings, together with recent results from CapRel^77^ and Avs2,^35^ suggest that protein-sensing defense systems may respond to more than one phage protein trigger, rather than a single specific target as previously thought.

It is becoming increasingly clear that bacteria have evolved dedicated strategies to counter jumbo phages, despite the sophisticated mechanisms these phages use to evade host defenses. The recently characterized Juk system directly targets the EPI vesicle, an early-stage evasion structure employed by jumbo phages.^27^ Our findings now show that Avs5 systems respond to proteins associated with or emerging from the EPI vesicle. In addition, our recent discoveries of the Ophion and Dionysus systems reveal parallel defense strategies that intercept jumbo phages at an early stage by targeting the EPI vesicle or preventing progression of the EPI vesicle to the phage nucleus.^78^ Together, these systems illustrate a broader strategy in which conserved phage countermeasures are used as vulnerabilities by bacterial immune defenses. It is likely that future studies will uncover additional defense systems specialized to target distinct stages of the jumbo phage lifecycle.

### Limitations of the study

While this study provides key insights into the activation and function of Avs5 systems, it also raises important questions. The precise mechanism by which PaAvs5 is activated by its phage-encoded trigger, JADA, remains unclear, in part because PaAvs5 was difficult to purify. Although we observed a rapid drop and subsequent recovery of NAD^+^ levels during infection, the mechanism underlying NAD^+^ restoration remains unknown. In addition, while JADA is clearly an essential activator of Avs5, its molecular function during phage infection remains to be elucidated.

## Resource availability

### Lead contact

Further information and requests for resources and reagents should be directed to and will be fulfilled by the lead contact, S.J.J.B. (stanbrouns@gmail.com).

### Materials availability

All unique bacterial strains, phages, and plasmids generated in this study are available from the lead contact without restriction.

### Data and code availability

• Liquid chromatography-mass spectrometry (LC-MS) data have been deposited at Zenodo as (doi: 10.5281/zenodo.15490963) and are publicly available as of the date of publication. Confocal microscopy data have been deposited at Mendeley data as (doi: 10.17632/sn5mvc6ygv.1) and are publicly available as of the date of publication. Shotgun proteomics raw data and search data obtained from enriched PAO1 with TurboID-PaAvs5 samples have been deposited in the PRIDE database (Proteome Exchange server ID: PXD065050). Cryo-EM maps were deposited under PDB accession code 9RP3 and EMDB accession code EMD-54139.
• This paper does not report original code.
• Any additional information required to reanalyze the data reported in this paper is available from the lead contact upon request.

## Acknowledgments

This work was supported by a grant from the European Research Council (ERC) CoG under the European Union’s Horizon 2020 research and innovation program (grant agreement no. 101003229) awarded to S.J.J.B. A.M. is supported by grants from Koningin Wilhelmina Fonds (KWF, grant agreement no. 15602) and Nederlandse Organisatie voor Wetenschappelijk Onderzoek (NWO, grant agreement no. OCENW.XS23.1.006). M.P. was supported by the Peter und Traudl Engelhorn Stiftung. B.E.C. was supported by the Swiss National Science Foundation. We acknowledge the infrastructure provided by the Kavli Nanolab Imaging Centre at TU Delft for optical microscopy. We thank Alexander Myasnikov, Bertrand Beckert, and Sergey Nazarov (Dubochet Center for Imaging, EPFL-UNIL-UNIGE, Switzerland) for assistance with cryo-EM data collection. We thank Prof. Rob Lavigne (Katholieke Universiteit Leuven, Belgium) for kindly providing plasmid pSTDesR, Franklin Nobrega (University of Southampton, United Kingdom) for providing strains and plasmids for BACTH, and Milan Gerovac (Helmholtz Centre for Infection Research, Germany) for providing phage PhiKZ.

## Author contributions

Conceptualization, A.M. and S.J.J.B.; methodology, A.M. and S.J.J.B.; formal analysis, A.M., A.R.C., D.F.v.d.B., and M. Pacesa; investigation, A.M., A.R.C., D.F., D.F.v.d.B., H.v.d.B., A.D.Z-D., A.R-.M., M. Pabst, and M. Pacesa; visualization, A.M., D.F.v.d.B., H.v.d.B., and M. Pacesa.; writing – original draft, A.M.; writing – review & editing, A.M., A.R.C., D.F., M. Pacesa, and S.J.J.B.; resources, A.M., M. Pacesa., B.E.C., and S.J.J.B.; supervision, A.M. and S.J.J.B.; funding acquisition, A.M. and S.J.J.B.

## Declaration of interests

The authors declare no competing interests.

## STAR★Methods

### Key resources table

**Table:** 

REAGENT or RESOURCE	SOURCE	IDENTIFIER
**Bacterial and virus strains**		

*P. aeruginosa* PAO1	Fagenbank	N/A
Clinical *P. aeruginosa* GCA_030069065.1	UMC Utrecht	GCA_030069065.1
Clinical *P. aeruginosa* MCO2951615.1	UMC Utrecht	MCO2951615.1
Clinical *P. aeruginosa* MCO2344054.1	UMC Utrecht	MCO2344054.1
NEB 5-alpha competent *E. coli*	New England Biolabs	Cat# C2987H
*E. coli* BTH101	Nobrega lab, University of Southampton, UK	Cat# EUB001 (Euromedex)
*E. coli* BL21-AI	Brouns Lab	N/A
vB_PaeM_FBPa2	Fagenbank	N/A
vB_PaeP_FBPa3	Fagenbank	N/A
vB_PaeP_FBPa6	Fagenbank	N/A
vB_PaeM_FBPa10	Fagenbank	N/A
vB_PaeM_FBPa12	Fagenbank	N/A
vB_PaeP_FBPa18	Fagenbank	N/A
vB_PaeM_FBPa21	Fagenbank	N/A
vB_PaeP_FBPa22	Fagenbank	N/A
vB_PaeM_FBPa24	Fagenbank	N/A
vB_PaeP_FBPa25	Fagenbank	N/A
vB_PaeS_FBPa28	Fagenbank	N/A
vB_PaeP_FBPa29	Fagenbank	N/A
vB_PaeM_FBPa33	Fagenbank	N/A
vB_PaeM_FBPa34	Fagenbank	N/A
vB_PaeM_FBPa35	Fagenbank	N/A
vB_PaeM_FBPa50	Fagenbank	N/A
vB_PaeS_FBPa53	Fagenbank	N/A
PP7	LGC Standards	ATCC-15692-B4

**Chemicals, peptides, and recombinant proteins**		

Adenosine 5′-Diphosphoribose Sodium	Sigma-Aldrich	Cat# A0752
Agar	Sigma-Aldrich	Cat# 05039
Agarose	Promega Corporation	Cat# V3125
Amicon® Ultra Centrifugal Filter, 3 kDa MWCO	Millipore	Cat# UFC500308
Amicon® Ultra Centrifugal Filter, 30 kDa MWCO	Millipore	Cat# UFC903008
Ampicillin	Carl Roth	Cat# K029.4
Biotin	Sigma-Aldrich	Cat# N8878-25G
BamHI	New England Biolabs	Cat# R0136S
β-Nicotinamide Adenine Dinucleotide Sodium Salt	Sigma-Aldrich	Cat# N0632
Carbenicillin	Fisher Scientific	Cat# BP2648-5
cOmplete™, EDTA-free Protease Inhibitor Cocktail	Sigma-Aldrich	Cat# 11873580001
Disruptor Beads 0.1mm	Scientific Instruments Inc	Cat# SI-BG01
Dithiothreitol	Sigma-Aldrich	Cat# 43815-5G
DpnI	New England Biolabs	Cat# R0176S
EcoRI	New England Biolabs	Cat# R0101S
FastAP	Fischer Scientific	Cat# EF0651
Glycerol	Fisher Scientific	Cat# 158920025
Isopropyl β-d-1-thiogalactopyranoside (IPTG)	Sigma-Aldrich	Cat# I5502
L-arabinose	Sigma-Aldrich	Cat# A3256-100
Lysogeny Broth (LB)	Sigma-Aldrich	Cat# L3022
Lysozyme	Sigma-Aldrich	Cat# L6876
Magnesium chloride	Fisher Scientific	Cat# AM9530G
MES SDS Running Buffer Powder	GenScript	Cat# M00677
Oasis HLB 96-well Plate	Waters	WAT058951
Phosphate buffered saline (PBS) tablets	Calbiochem	Cat# 524650-EA
Potassium chloride	Sigma-Aldrich	Cat# P3911-500G
Precision Plus Protein- Dual Xtra Prestained Protein Standards	Bio-Rad	Cat# 1610377
Q5 DNA polymerase	New England Biolabs	Cat# M0491L
Q5 High GC Enhancer	New England Biolabs	Cat# M4093
Rhamnose	Fisher Scientific	Cat# 10583731
Sequencing Grade Modified Trypsin	Promega	Cat# V5111
Sodium chloride	Fisher Scientific	Cat# AC424290010
Strep-Tactin XT 4Flow high-capacity resin	IBA Lifesciences	Cat# 2-5030-002
Streptomycin sulfate salt	Sigma Aldrich	Cat# S9137-25G
SurePAGE™, Bis-Tris, 10x8, 4%–20%, 10 wells	GenScript	Cat# M00655
T4 DNA Ligase	New England Biolabs	Cat# M0202S
TAE 40X	Promega Corporation	Cat# V4281
Tris base	Sigma-Aldrich	Cat# 10708976001

**Critical commercial assays**		

GeneJET Plasmid Miniprep Kit	Fisher Scientific	Cat# K0503
NEBuilder® HiFi DNA Assembly Master Mix	New England Biolabs	Cat# E2621L
Zymoclean Gel DNA Recovery Kit	Zymo Research	Cat# D4002

**Deposited data**		

Phylogenetic tree, multiple sequence alignments	This study	Zenodo: http://www.doi.org/10.5281/zenodo.15490963
Confocal microscopy raw data	This study	Mendeley Data: http://www.doi.org/10.17632/sn5mvc6ygv.1
Shotgun proteomics raw data	This study	PRIDE: PXD PXD065050
Cryo-EM maps	This study	PDB: 9RP3, EMDB: EMD-54139

**Oligonucleotides**		

All the DNA oligonucleotides are listed in Table S3	IDT	N/A

**Recombinant DNA**		

All plasmids are listed and described in Table S4	N/A	N/A
gBlock gene fragments are listed in Table S3	IDT	N/A

**Software and algorithms**		

AcquireMP	Refeyn Ltd.	N/A
Adobe Illustrator 26.0.1	Adobe	N/A
Alphafold 2	(Jumper et al. 2021)^79^	https://github.com/google-deepmind/alphafold
AlphaFold 3	(Abramson et al. 2024)^80^	https://alphafoldserver.com/
blastp v2.16.0	(Altschul et al. 1990)^81^	https://blast.ncbi.nlm.nih.gov/Blast.cgi
BFVD	(Kim et al. 2025)^71^	https://bfvd.foldseek.com
CD-SearchD v3.20	(Marchler-Bauer and Bryant 2004)^82^	https://www.ncbi.nlm.nih.gov/Structure/cdd/wrpsb.cgi
Cellpose-napari v0.2.0	(Stringer et al. 2021)^83^	https://github.com/MouseLand/cellpose-napari
ChimeraX	University of California, San Francisco (UCSF)	N/A
ColabFold v1.5.5	(Mirdita et al. 2022)^84^	https://colab.research.google.com/github/sokrypton/ColabFold/blob/main/AlphaFold2.ipynb
Coot v0.9.5	(Emsley et al. 2010)^85^	https://www2.mrc-lmb.cam.ac.uk/personal/pemsley/coot/
cryoSPARC v.4.6.2	(Punjani et al. 2017)^86^	https://cryosparc.com
DeepBacs	(Spahn et al. 2022)^87^	https://github.com/HenriquesLab/DeepBacs
DiscoverMP	Refeyn Ltd.	N/A
EPU v.2.12.1	Thermo Scientific	N/A
Foldseek v6	(van Kempen et al. 2023)^52^	https://github.com/steineggerlab/foldseek
GraphPad Prism 10.5	GraphPad	N/A
HHrepID v1.0	(Biegert and Söding 2008)^88^	https://toolkit.tuebingen.mpg.de/tools/hhrepid
Hmmbuild v3.4	(Finn et al. 2011)^89^	https://anaconda.org/bioconda/hmmer
InterProScan v5.60-92.0	(Jones et al. 2014)^90^	https://github.com/ebi-pf-team/interproscan
iQ-Tree2 v2.3.6	(Minh et al. 2020)^91^	http://www.iqtree.org/
iTOL v5.0	(Letunic and Bork 2021)^92^	https://itol.embl.de
LoVis4u v0.2.0	(Egorov and Atkinson 2025)^93^	https://github.com/art-egorov/lovis4u
MassHunter data acquisition software v10.1	Agilent	N/A
ModelAngelo v1.0.14	(Jamali et al. 2024)^94^	https://github.com/3dem/model-angelo
MolProbity v.4.5.1	(Davis et al. 2007)^95^	https://www.phenix-online.org/documentation/reference/molprobity_tool.html
Napari v0.6.2	Napari contributors 2019	10.5281/zenodo.3555620
NIS-Elements	Nikon Instruments Inc	N/A
PEAKS Studio X	Bioinformatics solutions Inc.	N/A
Phanotate v1.5.0	(McNair et al. 2019)^96^	https://github.com/deprekate/PHANOTATE
Pharokka v1.6.0	(Bouras et al. 2023)^97^	https://github.com/gbouras13/pharokka
Phenix.real_space_refine v.1.20.1-4487	(Afonine et al. 2018)^98^	https://www.phenix-online.org/documentation/reference/real_space_refine.html
Phold v0.2.0	N/A	https://github.com/gbouras13/phold
PSI-BLAST	(Altschul et al. 1997)^99^	https://blast.ncbi.nlm.nih.gov/Blast.cgi?PAGE_TYPE=BlastSearch&PROGRAM=blastp&BLAST_PROGRAMS=psiBlast
Skyline v25.0	(MacLean et al. 2010)^100^	https://skyline.ms/project/home/software/Skyline/begin.view
TPRpred v1.0	(Karpenahalli et al. 2007)^101^	https://toolkit.tuebingen.mpg.de/tools/tprpred

**Other**		

300-mesh holey carbon grid (Au 1.2/1.3)	Quantifoil Micro Tools	N/A
Acclaim PepMap RSLC RP C18 reverse phase, (75mm x 150mm, 2mm)	Thermo Scientific	Cat# 164568
ӒKTA pure™ chromatography system	Cytiva	N/A
Bio-Rad Gel Doc XR+	Bio-Rad	Cat# Bio-Rad Gel Doc XR+
Continuous flow cell disruptor CF1	Constant Systems	N/A
Epoch 2 microplate reader	Biotek Instruments	Cat# EPOCH2
EASY nano LC 1200	Thermo Scientific	N/A
FastPrep-25 5G bead beater	MP Biomedicals	Cat# 116005500
FEI Falcon IV detector	Thermo Scientific	N/A
Nanophotometer	Implen	Cat# NP80
Nikon A1R/SIM laser scanning confocal microscope	Nikon Instruments Inc	N/A
QE plus Orbitrap mass spectrometer	Thermo Scientific	N/A
Refeyn TwoMP	Refeyn	N/A
Superdex™ 200 Increase 10/300 GL	Cytiva	Cat# GE29219757
Superose™ 6 Increase 10/300 GL	Cytiva	Cat# 29091596
Titan Krios G4 microscope	Thermo Scientific	N/A
Triple Quadrupole LC/MS	Agilent	Cat# G6460C
Vitrobot	Thermo Scientific	N/A

### Experimental Model and Study Participant Details

#### Bacteria and Phages

Three clinical isolates of *Pseudomonas aeruginosa* (accession number: GCA_030069065.1, MCO2951615.1, MCO2344054.1) obtained from the University Medical Center Utrecht was utilized to amplify the PaAvs5 defense systems using PCR with suitable primers. For cloning experiments involving the PaAvs5 systems into plasmid pUCP20, *Escherichia coli* strain DH5α and *Pseudomonas aeruginosa* strain PAO1 were employed. All bacterial cultures were maintained in Lysogeny Broth (LB) at 37°C with shaking at 180 RPM or on LB agar (LBA, 1.5% agar w/v) plates at 37°C, unless specified otherwise. Strains containing plasmid pUCP20 were grown in LB media supplemented with either 100 μg/mL ampicillin (for *E. coli*) or 200 μg/mL carbenicillin (for *P. aeruginosa*).

The phages used in this study, except for *Pseudomonas* phage PP7 (obtained from LGC Standards), were obtained from the Fagenbank. Phages were propagated in liquid cultures with PAO1, followed by centrifugation at 3,000 × g for 15 minutes, filtration through 0.2 μm PES filters, and storage as lysates at 4°C until needed.

### Method Details

#### Phylogenetic Tree of Avs5

The phylogenetic tree of Avs5 was built with the Avs5 protein sequences provided by Gao et al. (2020).^30^ Moreover, Position-Specific Iterated BLAST (PSI-BLAST)^99^ was performed on the ClusteredNR (nr_cluster_seq) database of NCBI to search for additional Avs5 homologs. Human Sterile alpha motif domain-containing protein 9 (SAMHD9) (Genbank: NP_001180236.1) was used as a root for the Avs5 phylogenetic tree in the rooted version of the tree. Collectively, these 335 sequences were aligned using Muscle v5.1.0^102^ (default settings) and trimmed using trimAl v1.5.0 (default settings). The resulting trimmed alignment was used to build and bootstrap a phylogenetic tree using IQ-Tree2 v2.3.6^91^ (-B 1000, --alrt 1000, -m TEST). This phylogenetic tree was visualized using iTol v5.0.^92^

#### Annotation of Functional Domains

CDD v3.21^82^^,^^103^ and InterProScan v5^90^ were used to annotate the functional domains. Additionally, protein tandem repeat domains were searched for using TPRpred v1.0^101^ and HHrepID v1.0^88^ to detect TPR and de-novo tandem repeats, respectively. As well as a protein sequence and structural comparison using blastp v2.16.0^81^ all-versus-all and AlphaFold3.^79^^,^^80^

#### Cloning of PaAvs5 Systems into PAO1

The PaAvs5 systems and their promoter regions were amplified from *Pseudomonas aeruginosa* strains using the primers (Integrated DNA Technologies) listed in the Table S3 and Q5 High-Fidelity DNA Polymerase (New England BioLabs) supplemented with Q5 High GC Enhancer (New England BioLabs). The resulting PCR products mixed with loading dye were visualized on 1% agarose gels supplemented with SYBR Safe DNA Gel Stain (Invitrogen), and the target bands were excised and purified with the Zymoclean Gel DNA Recovery Kit (Zymo Research). Plasmid pUCP20 was digested with BamHI and EcoRI (New England BioLabs), treated with FastAP (Thermo Scientific) to remove any overhangs, and then purified using the Zymo DNA Clean & Concentrator Kit (Zymo Research). The amplified PaAvs5 systems were then cloned into a digested pUCP20 plasmid using the NEBuilder HiFi DNA Assembly Master Mix (New England BioLabs) and subsequently transformed into high-efficiency NEB 5-alpha competent *E. coli* (New England BioLabs), following the manufacturer's protocol. After growing the transformed *E. coli* in S.O.C. (Super Optimal broth with Catabolite repression) for 1 hour at 37°C, the culture is plated on LB agar supplemented with 100 μg/mL ampicillin and incubated overnight at 37°C.

Plasmids were extracted using the GeneJet Plasmid Miniprep Kit from overnight cultures from individual colonies from the transformed plate and verified through nanopore sequencing (Macrogen). Confirmed plasmids were introduced into *P. aeruginosa* strain PAO1 via electroporation. To prepare electrocompetent PAO1 cells, an overnight culture of PAO1 was centrifuged at 16,000 × g for two minutes at room temperature.^104^ The pellet was washed twice with 300 mM sucrose and resuspended in the same solution. For electroporation, 200–500 ng of plasmid DNA was added to 20 μl of the electrocompetent PAO1 suspension. The mixture was transferred to a 2.5 mm electroporation cuvette and electroporated at 2.5 kV. The transformants were then plated on LB agar supplemented with 200 μg/mL carbenicillin.

Point mutations in the Sir2 or ATPase domains, as well as deletions within the sensor or putative signal peptide domains of Avs5 in pUCP20, were introduced using round-the-horn site-directed mutagenesis with 5′ phosphorylated primers (Integrated DNA Technologies) and Q5 DNA Polymerase supplemented with Q5 High GC Enhancer (New England BioLabs). The resulting PCR products were digested with DpnI (New England BioLabs), separated on 1% agarose gels, and the bands were excised and purified using the Zymo Gel DNA Recovery Kit. The amplified plasmids were then ligated using T4 DNA ligase (New England BioLabs) at room temperature for two hours and transformed into chemically competent NEB 5-alpha Competent *E. coli* according to the manufacturer’s instructions.

Plasmids were extracted using the GeneJET Plasmid Miniprep Kit and verified by nanopore or Sanger sequencing (Macrogen). The confirmed plasmids were subsequently introduced into *P. aeruginosa* strain PAO1 via electroporation as described above. The transformants were then plated on LB agar supplemented with 200 μg/mL carbenicillin. List of constructed plasmids are provided in Table S4.

#### Cloning of PaAvs5-1 Fusion Proteins into PAO1

The gBlocks encoding mNeonGreen and TurboID (Integrated DNA Technologies) were amplified using primers with overhangs homologous to the PaAvs5-1 gene and the pUCP20 plasmid. The primer sequences are listed in Table S3. The amplified PaAvs5-1 constructs were cloned into the digested pUCP20 plasmid using the NEBuilder HiFi DNA Assembly Master Mix (New England BioLabs) and subsequently transformed into high-efficiency NEB 5-alpha competent *E. coli* (New England BioLabs). The transformed *E. coli* is plated on LB agar supplemented with 100 μg/mL ampicillin.

Plasmids were extracted using the GeneJET Plasmid Miniprep Kit and verified by sequencing (Macrogen). The confirmed plasmids were then introduced into PAO1 by electroporation as described above. The transformants were then plated on LB agar supplemented with 200 μg/mL carbenicillin.

For both mNeonGreen and TurboID, plasmids were constructed with the fusion at either the amino or carboxy terminus of PaAvs5-1. The effect of the fusion on PaAvs5 activity was evaluated using an efficiency-of-plating assay with the phage Pa36. Experiments were subsequently conducted with the construct that had minimal impact on PaAvs5 system activity.

#### Cloning of Phage Genes

Phage genes were PCR-amplified using Q5 DNA polymerase (New England BioLabs) supplemented with Q5 High GC Enhancer (New England BioLabs) with primers (Integrated DNA Technologies) containing overhangs homologous to the pSTDesR plasmid.^105^ Primer sequences are listed in Table S3. The amplified phage gene constructs were cloned into the PCR-amplified pSTDesR plasmid using NEBuilder HiFi DNA Assembly Master Mix (New England BioLabs) and subsequently transformed into high-efficiency NEB 5-alpha competent *E. coli* (New England BioLabs). The phage gene inserts are under the control of a rhamnose-inducible promoter, pRhaBAD. Transformed *E. coli* cells were plated on LB agar supplemented with 12.5 μg/mL streptomycin.

Plasmids were extracted using the GeneJET Plasmid Miniprep Kit and verified by sequencing (Macrogen). The confirmed plasmids were then introduced into PAO1 by electroporation as described above. The transformants were then plated on LB agar supplemented with 25 μg/mL streptomycin.

#### Efficiency of Plating

Phage stocks were diluted 10-fold serially in LB in a 96-well plate, and the resulting dilutions were spotted onto double-layer agar plates supplemented with 200 μg/mL carbenicillin and inoculated with *P. aeruginosa* PAO1. These plates contained either the empty pUCP20 plasmid, pUCP20 with PaAvs5-1, mutant PaAvs5-1, or PaAvs5-1 with a fusion protein, following the small plaque drop assay method. To assess the anti-phage activity of the defense systems, the fold reduction in phage infectivity was calculated by comparing the phage infectivity in the PAO1 strain carrying the defense systems encoded by pUCP20 to that in the strain carrying the empty plasmid.

#### Liquid Culture Collapse Assays

Overnight bacterial cultures were diluted to an OD_600_ of approximately 0.1 in LB and transferred into 96-well plates. The bacterial strains containing pUCP20 plasmids were supplemented with 200 μg/mL carbenicillin. Phages were added at multiplicities of infection (MOIs) of 0.01 and 1, and the plates were incubated at 37°C in an Epoch2 microplate spectrophotometer (Biotek) with double orbital shaking. OD_600_ measurements were taken every 10 minutes for 24 hours to monitor bacterial growth.

#### One-Step Growth Curve

*P. aeruginosa* PAO1 strains carrying either an empty vector or a plasmid encoding the defense system were cultured in LB medium to an optical density (OD_600_) of 0.3–0.4. Cells were harvested by centrifugation at 3,200 × *g* for 10 minutes and resuspended in fresh LB at half the original culture volume. Pa36 phage was added at a multiplicity of infection (MOI) of 0.01 and allowed to adsorb for 10 minutes at 37°C with shaking (180 rpm). Following adsorption, cells were pelleted again and resuspended in LB to restore the initial culture volume. Cultures were incubated at 37°C with shaking for 110 minutes, and aliquots were taken immediately after infection (time 0) and every 5 minutes thereafter. Each sample was serially diluted (10-fold series) and spotted onto PAO1 double-layer agar plates to determine phage titers (PFU/mL).

#### Cell Lysate Preparation for NAD^+^ and ADPR LC-MS

Overnight cultures of *P. aeruginosa* PAO1 strains containing the pUCP20 plasmid with PaAvs5-1, the PaAvs5-1 N110A point mutant, or no defense systems were diluted 1:100 in 250 mL LB and incubated at 37°C with shaking at 180 RPM. When the OD_600_ reached 0.3, 50 mL of uninfected culture (corresponding to time = 0 minutes) was removed. Phage stock was then added to the remaining culture to achieve a MOI of 3. The flasks were incubated at 37°C with shaking at 180 RPM for the duration of the experiment.

At each time point, 50 mL of the culture was removed. Immediately after sampling, the tubes were centrifuged at 4 °C for five minutes at 3900 RPM to stop the phage infection process. The supernatant was discarded, and the pellet was frozen at -20 °C. To extract metabolites and lysing the cells, 1 mL of 100 mM phosphate buffer (pH 8) consisting of Potassium Phosphate Dibasic (∼ 93.5 mM) and Potassium Phosphate Monobasic (∼ 6.5 mM) and supplemented with 4 mg/mL lysozyme was added to each pellet. The tubes were thawed on ice for ten minutes and then transferred to 2 mL FastPrep tubes (MP biomedicals) and 0.1 g of cell disruption media composed of 0.1 mm glass beads (Scientific Industries Inc) were added. To each tube, 0.5 μmol of β-Nicotinamide Adenine Dinucleotide Sodium Salt (Sigma) and Adenosine 5′-Diphosphoribose Sodium (Sigma) were added as internal standards. The cells were then lysed using the FastPrep-25 5G bead beater (MP biomedicals) for 40 seconds at the manufacturer's recommended settings for *E. coli*. The tubes were centrifuged at 4 °C for ten minutes at 15,000 × g to separate the beads from the lysate. The supernatant was then transferred to an Amicon Ultra-0.5 centrifugal filter unit (3 kDa) and centrifuged for one hour at 4°C at 6000 × g. The filtrate was collected, frozen at -20°C and subsequently used for LC-MS analysis.

#### Quantification of NAD± and ADPR by LC-MS

The LC-MS analysis was performed using an Agilent LC/MS system consisting of a high-pressure liquid chromatography set-up coupled to a triple-quadrupole (QQQ) mass spectrometer (G6460C) equipped with a standard electrospray ionization (ESI) source. Both systems were operated through MassHunter data acquisition software (version 10.1). 1 μL of each sample was injected into the column of the HPLC. NAD^+^ and ADPR were delivered to a CSH C18 guard column and a CSH C18 column (Waters) (2.1 mm by 50 mm, 1.7-μm pore size) at 30°C with a flow rate of 0.3 mL/min using the following binary gradient: 0% B (ACN, 25 mM FA), ramp to 85% B in 9 min, followed by a 30 sec hold at 85% B, a 2 min ramp back to 0% B, and a 3 min re-equilibration (A, 20 mM ammonium formate). Next, the metabolites were eluted from the column, and the eluent was sprayed into the mass spectrometer, which was operated in data-dependent mode, specifically in dynamic multiple-reaction monitoring (dMRM) mode using transitions. Each MRM transition was generated by optimizing the fragmentor voltage and the collision energy. The dMRM was acquired in positive mode with a cycle time of 500 ms. Data processing was done using Skyline.^100^

For the quantification of NAD^+^ and ADPR, a calibration curve (0–500 μM) using the standard addition method was employed. The batch design for running the samples was as follows: first, the calibration curve was analysed, followed by a blank sample. Then, the randomized samples were run, with a blank injected after every five samples to monitor carry-over. Subsequently, the calibration curve was injected again.

In Skyline, the peaks corresponding to NAD^+^ and ADPR were integrated for quantification, and the areas under the curves were exported for further analysis. A linear calibration curve (R^2^ > 0.9) was obtained for concentration calculations of each compound in all the samples.

#### Confocal Fluorescence Microscopy of PaAvs5-1

Exponentially growing PAO1 cultures (OD_600_ ≈ 0.3) containing either the fluorescently tagged PaAvs5-1 or negative were infected with phage at an MOI ≥3 so that all bacteria are infected. The phage was allowed to adsorb for 10 minutes at 37°C. The cells were centrifuged at 9,000g for 1 minute. The cell pellet was resuspended in 100 μL of LB. 2 μL each of 4',6-Diamidino-2-Phenylindole (DAPI, 5 mg/mL) and MitoTracker Deep Red FM (5 mg/mL) were added to the cell suspension. The resuspended cells (2 μL) were spotted onto 1% agarose pads.

Visualization was performed using a Nikon A1R/SIM laser scanning confocal microscope (inverted Nikon Ti Eclipse body) equipped with a 100× oil immersion objective (SR Apo TIRF; numerical aperture 1.49). Lasers were used sequentially to excite the different channels, from longest to shortest wavelength: MitoTracker Deep Red FM with 640 nm (emission filter: 700/75 nm), mNeonGreen with 488 nm (emission filter: 525/50 nm), and DAPI with 405 nm (emission filter: 450/50 nm), all passed through a 405/488/543/640 excitation dichroic. For JADA–mScarlet3 imaging, excitation was performed using a 561 nm laser with a Texas Red emission filter (emission filter: 595/50 nm). Z-stacks were acquired using a Nikon A1 Piezo Z Drive at intervals of either 0.2 or 0.1 μm (10–20 slices), capturing different planes of all bacteria within the field of view (512 × 512 pixels, corresponding to 36.79 × 36.79 μm, satisfying Nyquist criteria). A pinhole size corresponding to 1.2 AU referenced to the shortest wavelength was used. Images were acquired at 12-bit depth using a Galvano scanner with Nikon NIS-Elements software. Image analysis was performed using Fiji, and Napari. To perform image analysis, bacteria were segmented using Cellpose^83^ using Deepbacs^87^ model in Napari. Presence of PaAvs5 or phage DNA foci and nucleus were determined from maximum intensity projections of the respective z stacks.

#### Proximity Labelling to Identify PaAvs5 Activator

The TurboID-PaAvs5 construct was cloned into the pUCP20 plasmid and introduced into PAO1 cells. The PaAvs5 system was N-terminally tagged with TurboID, as this configuration retained its defense phenotype against phage Pa36. In contrast, C-terminal tagging abolished the defense activity. Cultures were grown overnight and subsequently diluted to an OD_600_ of 0.1 in fresh medium supplemented with 500 μM biotin. Upon reaching an OD_600_ of 0.3, cells were infected with phage Pa36 at a MOI of 3.

#### Cell Harvesting, Protein Extraction, On Beads Enrichment and Proteolytic Digestion

At 20 minutes post-infection, cultures were harvested by centrifugation at 9000 g for 10 minutes at 4 °C. Cell pellets were stored at −80 °C until further processing.

For protein extraction, pellets were resuspended in lysis buffer (100 mM Tris-HCl pH 7.5, 150 mM NaCl, 5% glycerol, 1 mM DTT) and lysed using a sonication. Cell debris and beads were removed by centrifugation, and only the supernatant was retained. Biotinylated proteins were enriched via affinity purification using Strep-Tactin XT beads. Beads were washed three times with lysis buffer and retained for on-bead processing.

Enriched proteins were reduced with 10 mM dithiothreitol (DTT) in 100 mM ammonium bicarbonate (ABC) for 60 minutes at 37 °C with shaking at 300 rpm, followed by alkylation with 20 mM iodoacetamide (IAA) in 100 mM ABC for 30 minutes at room temperature in the dark. Proteins were then digested on-bead overnight (∼18 hours) at 37 °C with Trypsin Sequencing Grade, also at 300 rpm.

Following digestion, beads were removed by centrifugation, and the peptide-containing supernatant was subjected to solid-phase extraction using an Oasis HLB 96-well μElution Plate. The plate was conditioned with methanol, equilibrated with LC-MS grade water, and samples were loaded and washed twice with 5% methanol. Peptides were eluted sequentially with 200 μL of 2% formic acid in 80% methanol followed by 200 μL 1 mM ABC in 80% methanol. The combined eluates were dried using a SpeedVac evaporator at 45 °C for 3–4 hours.

Prior to LC-MS analysis, samples were resuspended in 15 μL of 3% acetonitrile + 0.01% trifluoroacetic acid (TFA) in LC-MS grade water and analyzed using a nanoLC-Q Exactive Plus Orbitrap system.

#### Shotgun Proteomic Analysis

An aliquot of each sample was analyzed using a nano-liquid-chromatography system consisting of an EASY nano LC 1200 equipped with an Acclaim PepMap RSLC RP C18 reverse phase column (75 mm x 150 mm, 2 mm) coupled to a QE plus Orbitrap mass spectrometer (Thermo Scientific, Germany). Solvent A was H_2_O containing 0.1% formic acid, and solvent B consisted of 80% acetonitrile in H_2_O, containing 0.1% formic acid. The flow rate was maintained at 350 nL/min. The Orbitrap was operated in top 10 data dependent acquisition (DDA) mode, acquiring peptide signals form 350–1250 m/z, at 70K resolution in MS1 with an AGC target of 3e6 and max IT of 100 ms. An aliquot of approx. 100 ng protein digest was analyzed using a linear gradient from 2% to 40% B over 60 minutes. MS2 acquisition was performed at 17.5K resolution, with an AGC target of 5e5, and a max IT of 100 ms, using a NCE of 28. Unassigned, singly charged as well as >4 charged mass peaks were excluded.

#### Database Searching of Mass Spectrometric Raw Data

Mass spectrometric raw data were processed using PEAKS Studio X (Bioinformatics Solutions Inc., Canada) for database searching and de novo sequencing. Database searching was performed using the reference proteome of Pseudomonas aeruginosa (UP000002438, strain ATCC 15692) including phage sequences, and the cRAP proteome (https://www.thegpm.org/crap/), allowing 20 ppm parent ion and 0.02 Da fragment mass error and up to 3 missed cleavages. Carbamidomethylation was set as fixed and Biotinylation (+ 226.08 Da) as variable modification. Database search further used decoy fusion for estimation of false discovery rates (FDR) and subsequent filtering of peptide spectrum matches for 1% FDR, and 2 unique peptides per protein.

#### Validation of Activators of PaAvs5

Overnight cultures of P. aeruginosa PAO1 strains harboring either wild-type PaAvs5-1 or the catalytically inactive dSir2 mutant (N110A), both expressed from the pUCP20 plasmid, were grown and made electrocompetent as described above. Candidate phage genes, cloned into pSTDesR plasmids under the control of a rhamnose-inducible promoter (100 ng/μL), were electroporated into these electrocompetent cells using the same electroporation protocol.

Following transformation, 1 mL of LB medium was added, and cells were incubated at 37 °C for 1 hour. Cultures were then centrifuged, and the pellets were resuspended in 200 μL of LB. Each transformation was tested on both induced and uninduced conditions by spotting twofold serial dilutions (5 μL) onto LB agar plates containing carbenicillin and streptomycin, either without inducer or supplemented with 2.5 mM rhamnose. Each transformation was performed in duplicate. Plates were allowed to dry, then incubated overnight at 37 °C. Colony-forming units were counted the next day, and activation was assessed by comparing colony numbers in the wild-type versus dSir2 mutant.

#### Bacterial Two-Hybrid Assay

The bacterial two-hybrid assay (Euromedex) was used to assess protein–protein interactions. The T25 fragment of *Bordetella pertussis* adenylate cyclase was fused to the C terminus of PaAvs5-1, and the T18 fragment was fused to either the N or C terminus of candidate genes. Constructs were generated using primers listed in Table S3. *E. coli* BTH101 cells (*F-, cya-99, araD139, galE15, galK16, rpsL1 (str r), hsdR2, mcrA1, mcrB1*) were streaked on LB agar containing 25 μg/ml streptomycin, 1 mM IPTG and 30 μg/mL X-Gal to isolate white colonies before transformation. Electrocompetent cells were prepared as described previously for *E. coli*, and plasmids were introduced by electroporation.

Co-transformants carrying T18 and T25 fusion plasmids, or control vectors encoding the GCN4 leucine zipper, were cultured in LB medium supplemented at 37°C with shaking for one hour. These cultures were spotted onto LB agar containing 1 mM IPTG, 30 μg/mL X-Gal, 50 μg/ml kanamycin and 100 μg/ml ampicillin, followed by incubation at 30°C for up to 48 hours before imaging.

#### JADA Locus Visualization

We downloaded the genomes of bacteriophages classified under the family Chimalliviridae as described in Prichard et al. (2023).^106^ Genome annotation was performed using Phannotate^96^, Pharroka,^97^ and Phold. To identify homologs of the JADA protein, we constructed a hidden Markov model (HMM) profile based on PSI-BLAST^99^ hits with >60% query coverage. Sequences were aligned using Muscle v5.1.0,^102^ and the HMM profile was built using hmmbuild.^89^ The genomic locus of JADA homologs was visualized using LoVis4u.^93^

#### JADA Protein Purification

The JADA gene (Pa36 gp316) was amplified from *Pseudomonas aeruginosa* phage Pa36 genomic DNA using Q5 polymerase (NEB) with primers designed to incorporate homology arms matching the pACYCDuet-1 vector. The PCR product was cloned into the vector via Gibson assembly, placing a N-terminal 6×His tag for affinity purification. The resulting plasmid was confirmed by nanopore sequencing and transformed into E. coli BL21-AI cells. They were then plated on chloramphenicol-containing LB agar plates.

For expression, these cells were inoculated in LB medium supplemented with chloramphenicol and grown at 37 °C until the culture reached an OD_600_ of 0.6. Cultures were then cold-shocked on ice for 1 hour and induced with 1 mM IPTG and 0.2% arabinose. Following induction, cells were transferred to 20 °C and incubated overnight with shaking.

Cells were harvested by centrifugation using an Avanti J-26 XP centrifuge at 3900 × g for 30 minutes at 4 °C. The supernatant was discarded, and the cell pellet was resuspended in ice-cold PBS. This suspension was centrifuged again under the same conditions, and the supernatant was removed. The resulting washed pellets were stored at –80 °C until further processing.

Pellets were thawed on ice and resuspended in lysis buffer (100 mM Tris-HCl pH 7.5, 300 mM NaCl, 1 mM DTT, 5% glycerol) supplemented with cOmplete protease inhibitor cocktail (Roche). Cell lysis was performed at 1 bar using a continuous flow cell disruptor (Constant Systems), and lysates were clarified by centrifugation in an Avanti centrifuge at 16000 × g for 30 minutes at 4 °C. The resulting supernatant was filtered through a 0.45 μm syringe filter prior to affinity purification.

For affinity purification, the lysate was loaded onto a Ni-NTA His-Select column (pre-equilibrated with ice cold lysis buffer supplemented with 25 mM imidazole). The column was washed with 10 column volumes of the same buffer, and the bound protein was eluted with lysis buffer containing 250 mM imidazole. Eluted fractions were concentrated and further purified by size exclusion chromatography using a Superdex 200 Increase 10/300 GL column on an ÄKTA system, with lysis buffer as the running buffer. The purified JADA protein was snap-frozen in liquid nitrogen and sent for cryo-EM analysis.

Prior to cryo-EM, thawed proteins (JADA alone, PaAvs5-1 alone, or mixture) were purified once more by size exclusion chromatography using a Superose 6 Increase column.

#### Mass Photometry of JADA

The oligomeric state of purified JADA was analyzed by mass photometry using a Refeyn TwoMP instrument (Refeyn Ltd.). Proteins were diluted to a final concentration of 200–400 nM in gel filtration buffer prior to measurement. Microscope coverslips were sequentially cleaned with 50% isopropanol in Milli-Q H₂O, followed by alternating rinses with 100% isopropanol and Milli-Q H₂O, and subsequently dried using compressed nitrogen gas. Cleaned coverslips were assembled with silicone gaskets prior to measurement and placed on the objective lens with a drop of Immersol immersion oil (Zeiss). Focus was adjusted using AcquireMP software (Refeyn) with buffer alone before adding the protein sample. For calibration, the MassFerence P1 protein standard (Refeyn; 86, 172, and 256 kDa) was measured under identical conditions to generate a contrast–mass calibration curve. Subsequently, JADA samples were mixed 1:10 with buffer (100 mM Tris-HCl pH 7.5, 300 mM NaCl), and movies were recorded for 60 seconds. The buffer used for mass photometry measurements did not contain DTT or glycerol, as these components interfere with accurate mass photometry readings. Ratiometric contrast values were converted to molecular masses using DiscoverMP software (Refeyn), and Gaussian fitting was applied to determine the relative abundance of oligomeric species.

#### JADA Cryo-EM Structure Determination

Purified JADA protein was applied to a glow-discharged 300-mesh holey carbon grid (Au 1.2/1.3, Quantifoil Micro Tools), blotted for 4 s at 95% humidity, 10 °C, plunge-frozen in liquid ethane (Vitrobot, Thermo Fisher Scientific) and stored in liquid nitrogen. Data collection was performed with automation program EPU (Thermo Fisher Scientific, v.2.12.1) on a 300 kV FEI Titan Krios G4 microscope equipped with a FEI Falcon IV detector. A total of 15624 micrographs were recorded with a pixel size of 0.726 Å. Acquired cryo-EM data was processed using cryoSPARC (v.4.6.2).^86^ Gain-corrected micrographs were imported, and micrographs with a resolution estimation worse than 6 Å were discarded after patch contrast transfer function estimation. Initial particles were picked using a blob picker with 70–105 Å particle size. Particles were extracted with a box size of 280 × 280 pixels, down sampled to 100 × 100. After two-dimensional classification, clean particles were used for ab initio three-dimensional reconstruction. After several rounds of three-dimensional classification, the class with most detailed features was reextracted using full box size and subjected to non-uniform and local refinement to generate high-resolution reconstructions. The local resolution was calculated and visualized using ChimeraX (v.1.9, UCSF).

For structure building, we used an initial model generated by ModelAngelo,^94^ which was then used as template input for ColabFold^84^ reprediction. Full dimer model was then fitted into density and manually refined using Coot (v0.9.5)^85^ Atomic model refinement was performed using Phenix.real_space_refine (v.1.20.1-4487).^98^ The quality of the refined model was assessed using MolProbity (v.4.5.1).^95^ The refined atomic models and corresponding cryo-EM maps were deposited under PDB accession code 9RP3 and EMDB accession code EMD-54139. Details of data collection and refinement statistics are shown in Table 1.

**Table 1:** Cryo-EM data collection, processing, and model refinement statistics for JADA/gp316 homodimer (PDB: 9RP3, EMDB: 54139)

**Data collection and processing**	
Voltage (kV)	300
Electron exposure (e–/Å^2^)	50.0
Defocus range (μm)	−0.8–2.0
Pixel size (Å)	0.726
Symmetry imposed	C1
Initial particle images (no)	2,351,876
Final particle images (no)	1,723,569
Map resolution (Å)	2.12
FSC threshold	0.143
Map sharpening *B* factor (Å^2^)	−78.0

**Model composition**	
Non-hydrogen atoms	6349
Protein residues	799
Ligands	0
ADP B factors (Å^2^)Protein	94.1
Ligand	-

**Root-mean-square deviations**	
Bond lengths (Å)	0.003
Bond angles (°)	0.664

Validation	
MolProbity score	1.46
Clashscore	8.52
Poor rotamers (%)	0.72

**Ramachandran plot**	
Favored (%)	98.74
Allowed (%)	1.26
Disallowed (%)	0.00

#### PaAvs5-1 Protein Purification

Expression and purification of PaAvs5-1 were performed using procedures similar to those described above for JADA. The gene was amplified with homology to the p13SS expression vector and cloned via Gibson assembly. The construct included a N terminal Twin-Strep-SUMO tag for affinity purification and was expressed in *E. coli* BL21-AI using streptomycin selection. Additional variants were also tested: (1) a codon-optimized version of PaAvs5-1 for *E. coli* expression; (2) a version cloned with a N-terminal 6×His tag in the pACYCDuet-1 backbone (as used for JADA); and (3) an N110A point mutant of PaAvs5-1.

For Twin-Strep–SUMO-tagged constructs, purification was carried out using StrepTactin resin. After cell lysis (see above for buffer and disruption method), the cleared lysate was passed over StrepTactin beads, washed with lysis buffer (100 mM Tris-HCl pH 7.5, 300 mM NaCl, 1 mM DTT, 5% glycerol), and eluted in the presence of 50 mM biotin. Expression was verified using SDS-PAGE and then purified further using size exclusion chromatography as stated above.

Despite testing multiple expression constructs and purification strategies, including the addition of ATP or the non-hydrolysable analog AMP-PNP, PaAvs5 consistently yielded very low amounts of protein. Attempts to visualize particles by cryo-EM, either for PaAvs5 purified alone with low yield or in combination with JADA, failed to produce any visible particles.

#### Pulldown of JADA with PaAvs5-1

Pulldown experiments were performed using refined expression constructs developed after initial attempts with PaAvs5-1 purification. JADA was cloned into the p2A-T expression plasmid without an affinity tag, while PaAvs5-1 dSir2 (N110A) was cloned into the p13SS vector with a C-terminal Twin-Strep tag. Both proteins were co-expressed in *E. coli* BL21-AI under standard expression conditions described above.

Cells were lysed as outlined for JADA purification. The cleared lysate was supplied to StrepTactin resin, washed with lysis buffer (100 mM Tris-HCl pH 7.5, 150 mM NaCl, 1 mM DTT, 5% glycerol), and eluted with 50 mM biotin. Eluted fractions were analyzed by SDS-PAGE. Under these conditions, JADA co-eluted with Twin-Strep–tagged PaAvs5-1 dSir2, indicating successful pulldown.

### Quantification And Statistical Analysis

Unless stated otherwise, experiments were performed in biological triplicates and are represented as the mean and standard deviation. Biological replicates are from cultures derived from individual bacterial colonies and treated as per the details described in the results and figure legends. Data were plotted in GraphPad Prism and all details are provided in the legends.

## Supplemental information

Document S1. Figures S1–S7

Table S1. Excel sheet containing the list of Avs5 homologs and associated metadata used for phylogenetic analysis, related to Figure 1A

Table S2. Excel sheet of protein IDs and annotations for TurboID interactome screen,related to Figure 5B

Table S3. List of primers used in this study, related to STAR Methods

Table S4. List of plasmids used in this study, related to STAR Methods

Document S2. Article plus supplemental information
