# Supplementary material for: Molecular basis for anti-jumbo phage immunity by AVAST type 5
Source: Mol Cell. 2026 Feb 19;86(4):740–756.e9. doi: 10.1016/j.molcel.2026.01.004 (PMC12931631; doi:10.1016/j.molcel.2026.01.004)
Supplement: Document S1. Figures S1–S7 [file mmc1.pdf]

Molecular Cell, Volume *86*

Supplemental information

Molecular basis for anti-jumbo phage immunity by AVAST type 5

Aswin Muralidharan, Ana Rita Costa, Desi Fierlier, Daan Frits van den Berg, Halewijn van den Bossche, Adja Damba Zoumaro-Djayoon, Alicia Rodríguez-Molina, Martin Pabst, Martin Pacesa, Bruno E. Correia, and Stan J.J. Brouns

Molecular basis for anti-jumbo phage immunity by AVAST type 5

Aswin Muralidharan, Ana Rita Costa, Desi Fierlier, Daan Frits van den Berg, Halewijn van den Bossche, Adja Damba Zoumaro-Djayoon, Alicia Rodríguez-Molina, Martin Pabst, Martin Pacesa, Bruno E. Correia, Stan J. J. Brouns

List of contents

Figure S1 Sequence Conservation and Functional Analysis of Avs5 Domains and Motifs, Related to Figure 1.

Figure S2 Fluorescence Microscopy of PaAvs5-1 and Domain Mutants, Related to Figure 4.

Figure S3: Localization of PaAvs5-1 During Pa34 Infection, Related to Figure 4.

Figure S4 AlphaFold3 Cofolding Predictions to Identify Potential Interactors of PaAvs5-1, Related to Figure 5.

Figure S5 Pulldown of JADA by Twin-Strep–tagged PaAvs5-1 dSir2, Related to Figure 5.

Figure S6 Conservation and Genomic Context of JADA Homologs Across Jumbo Phages, Related to Figure 6.

Figure S7 Cryo-EM Map Quality and Resolution Estimation of JADA Homodimer and Fluorescence microscopy of JADA, Related to Figure 6.

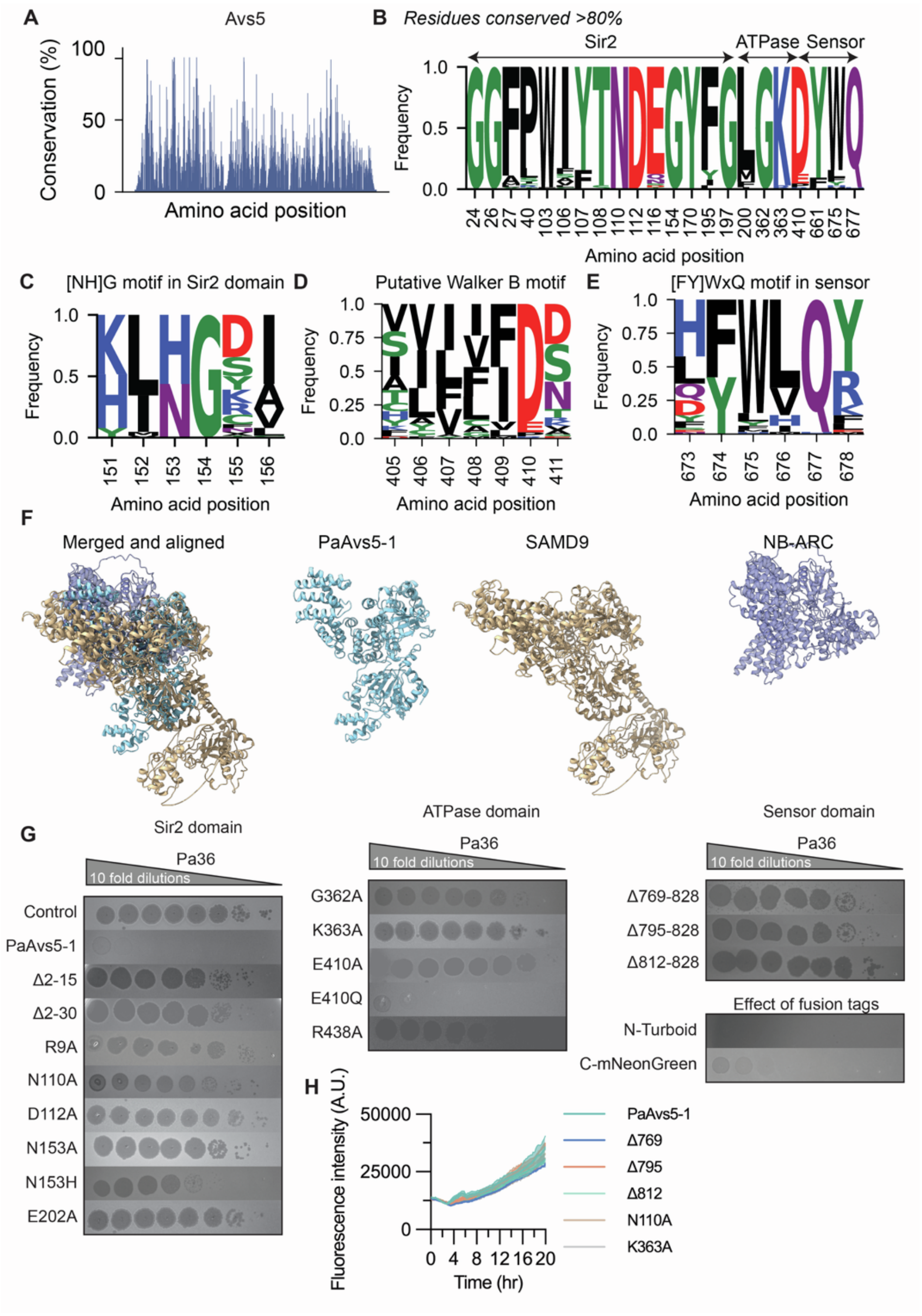

Figure S1: Sequence Conservation and Functional Analysis of Avs5 Domains and Motifs, Related to Figure 1.

(A) Plot showing percent amino acid conservation across 64 Avs5 homologs, based on multiple sequence alignment.

(B) Sequence logo highlighting residues conserved in >80% of homologs. Amino acid positions are numbered according to PaAvs5-1. Conserved regions span the Sir2, ATPase, and Sensor domains.

(C–E) Sequence logos of conserved motifs within functional domains of PaAvs5-1: (C) [NH]G motif in the Sir2 domain; (D) Putative Walker B motif in the ATPase domain; (E) [FY]WxQ motif in the sensor domain.

(F) Structural comparison of PaAvs5-1 with Foldseek-identified homologs from other organisms. The predicted structure of *Pa*Avs5-1 (cyan) shows strong similarity to the Sterile alpha motif domain-containing protein 9 (SAMD9; tan, UniProt: Q5K651) from *Homo sapiens* (Foldseek BFMD probability = 1, E-value = 4.6×10^{-16}, query position = 3–818, TM-score = 0.41, RMSD = 13.6) and to an NB-ARC domain-containing protein (purple, UniProt: A0A1C1CV94) from *Cladophialophora carrionii* (Foldseek probability = 1, E-value = 3.7×10^{-6}, query position = 199–825, TM-score = 0.29, RMSD = 25.7).

(G) Efficiency of plaquing (EOP) assay in *Pseudomonas aeruginosa* strain Pa36 for wild-type PaAvs5-1 and a panel of domain truncations, point mutants, and fluorescent fusion variants. Tenfold serial dilutions of phage lysate were spotted to assess antiviral activity.

(H) Fluorescence of C-terminally mNeonGreen-tagged PaAvs5-1 wild-type and domain mutants was monitored during growth. Comparable fluorescence intensities were observed across all constructs, indicating similar protein expression levels.

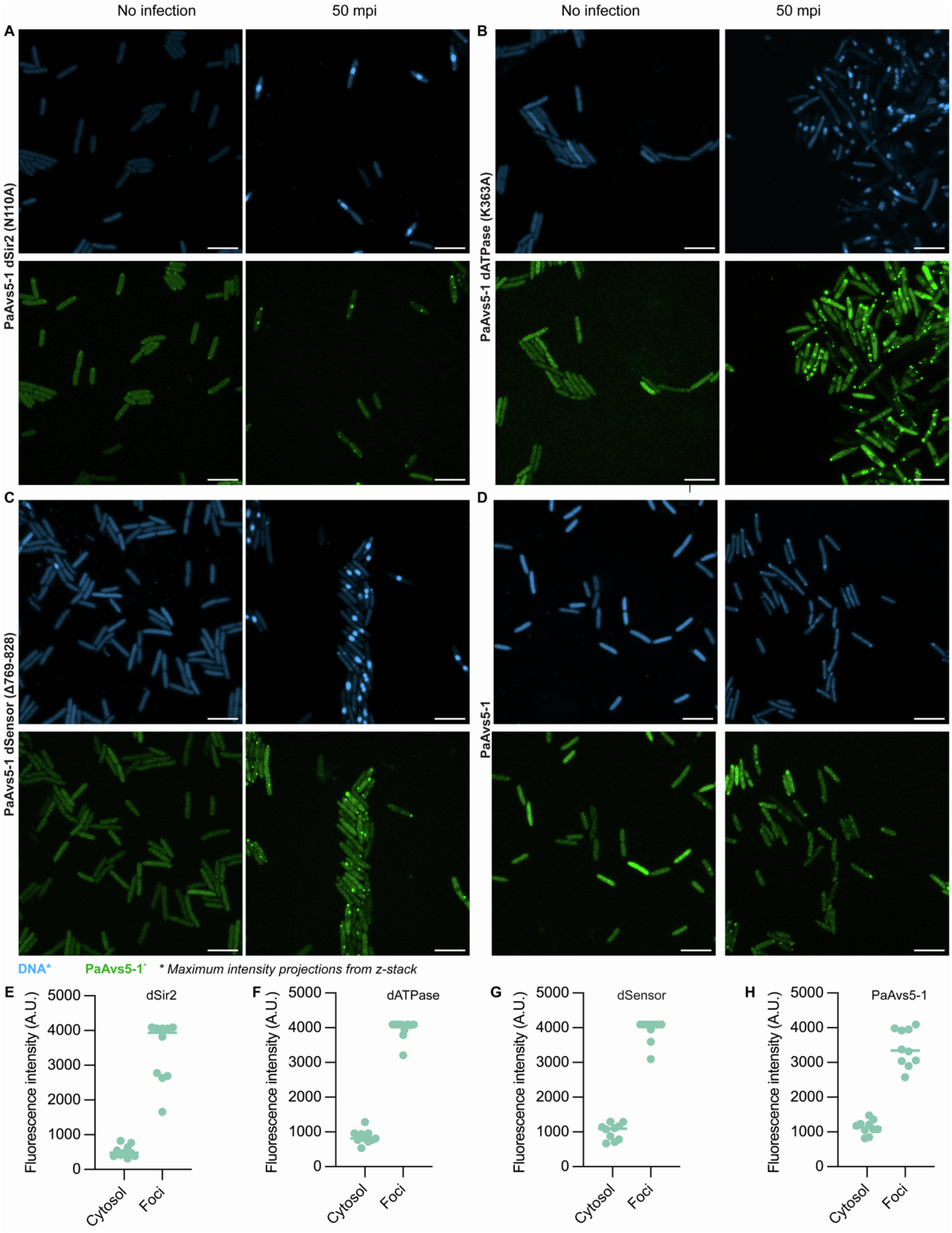

Figure S2: Subcellular Localization of PaAvs5-1 and Domain Mutants During Phage Infection, Related to Figure 4.

(A–D) Maximum intensity projection confocal fluorescence microscopy images of *P. aeruginosa* expressing wild-type or mutant PaAvs5-1 variants at indicated time points post-infection. DNA is stained in blue (DAPI), and PaAvs5-1 or its domain mutants are fused to a fluorescent tag and shown in green. (A) PaAvs5-1 dSir2 (N110A), (B) PaAvs5-1 dATPase (K363A), (C) PaAvs5-1 dSensor (Δ769–828), (D) Full-length PaAvs5-1. Scale bars represent 5 μm.

(E–H) Quantification of fluorescence intensity in the cytosol versus PaAvs5-1 foci (green channel). Ten randomly selected cells or foci were measured per sample. (E) dSir2, (F) dATPase, (G) dSensor, (H) Full-length PaAvs5-1. Maximum fluorescence intensity of 4095 AU indicates saturation.

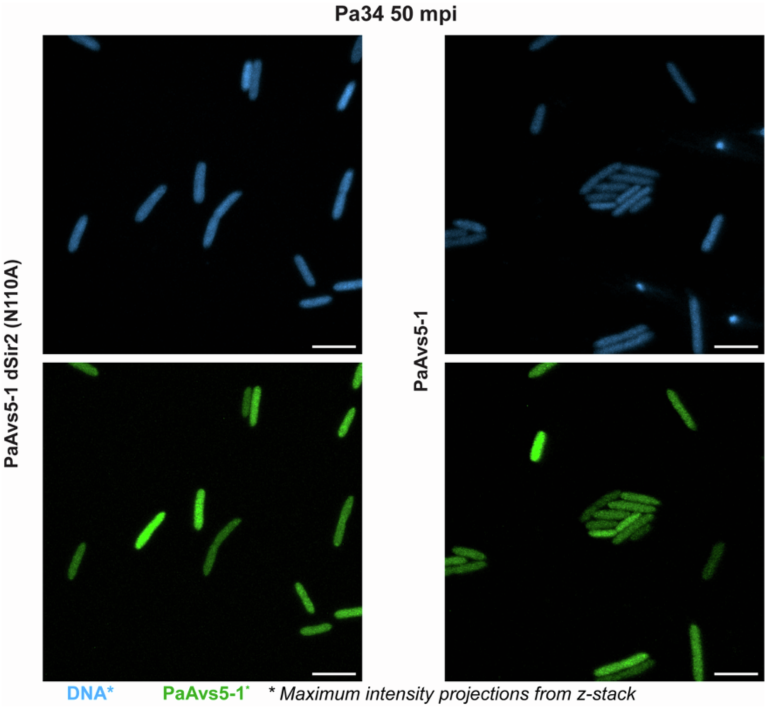

Figure S3: Localization of PaAvs5-1 During Pa34 Infection, Related to Figure 4.

Confocal fluorescence microscopy images of *P. aeruginosa* expressing PaAvs5-1 or its catalytically inactive mutant (dSir2 N110A), 50 minutes post-infection (mpi) with phage Pa34. DNA is shown in blue (DAPI), and PaAvs5-1 is shown in green. Unlike during Pa36 infection, PaAvs5-1 fails to form discrete foci during Pa34 infection, consistent with its inability to provide immunity against this phage. Scale bars: 5 μm.

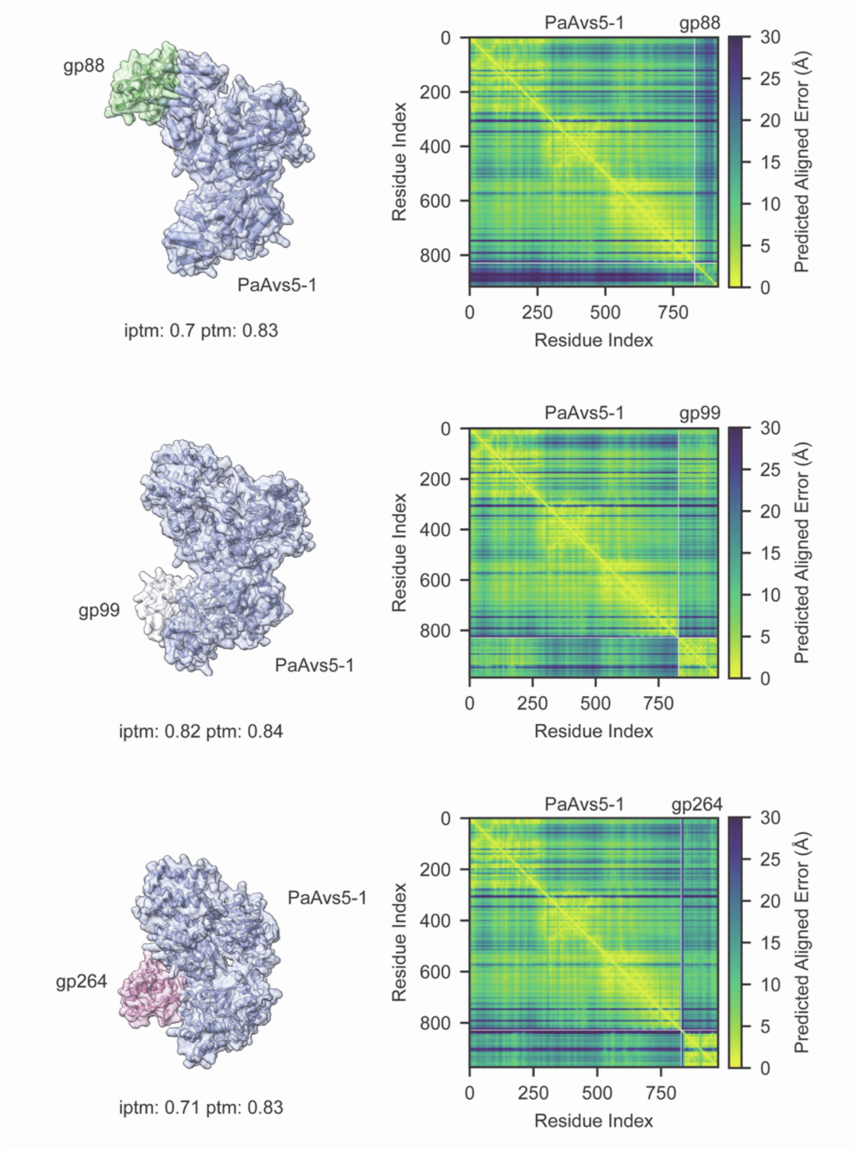

Figure S4: AlphaFold3 Cofolding Predictions to Identify Potential Interactors of PaAvs5-1, Related to Figure 5.

Structural predictions (left) and predicted aligned error (PAE) matrices (right) for PaAvs5-1 cofolded with three phage proteins from *Pseudomonas aeruginosa* phage Pa36 genome: gp88 (top), gp99 (middle), and gp264 (bottom). All 356 predicted phage proteins were tested using AlphaFold3, and these three candidates showed inter-protein TM (iptm) scores above 0.7, indicating possible stable interfaces. PAE plots reflect model confidence, with lower values (yellow) indicating higher accuracy in residue-residue alignment.

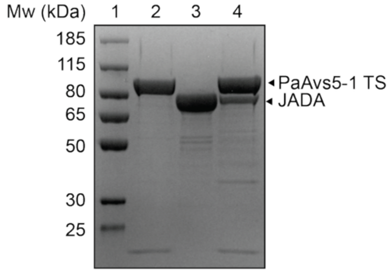

1. PageRuler plus ladder
2. PaAvs5-1 Twin-Strep
3. 6xHis JADA
4. PaAvs-1 dSir2 Twin-Strep + JADA

Figure S5: Pulldown of JADA by Twin-Strep–tagged PaAvs5-1 dSir2, Related to Figure 5.

SDS–PAGE analysis of the optimized pulldown assay. Lane 1 contains the molecular weight marker. Lane 2 shows PaAvs5-1 dSir2(N110A) purified alone using Strep-Tactin affinity chromatography. Lane 3 contains purified 6×His-tagged JADA (reference control). Lane 4 shows the eluate from Strep-Tactin affinity purification of PaAvs5-1 dSir2(N110A) co-expressed with untagged JADA. In lane 4, both PaAvs5-1 dSir2 and JADA are recovered, indicating that untagged JADA co-purifies with the Twin-Strep–tagged Avs5 variant.

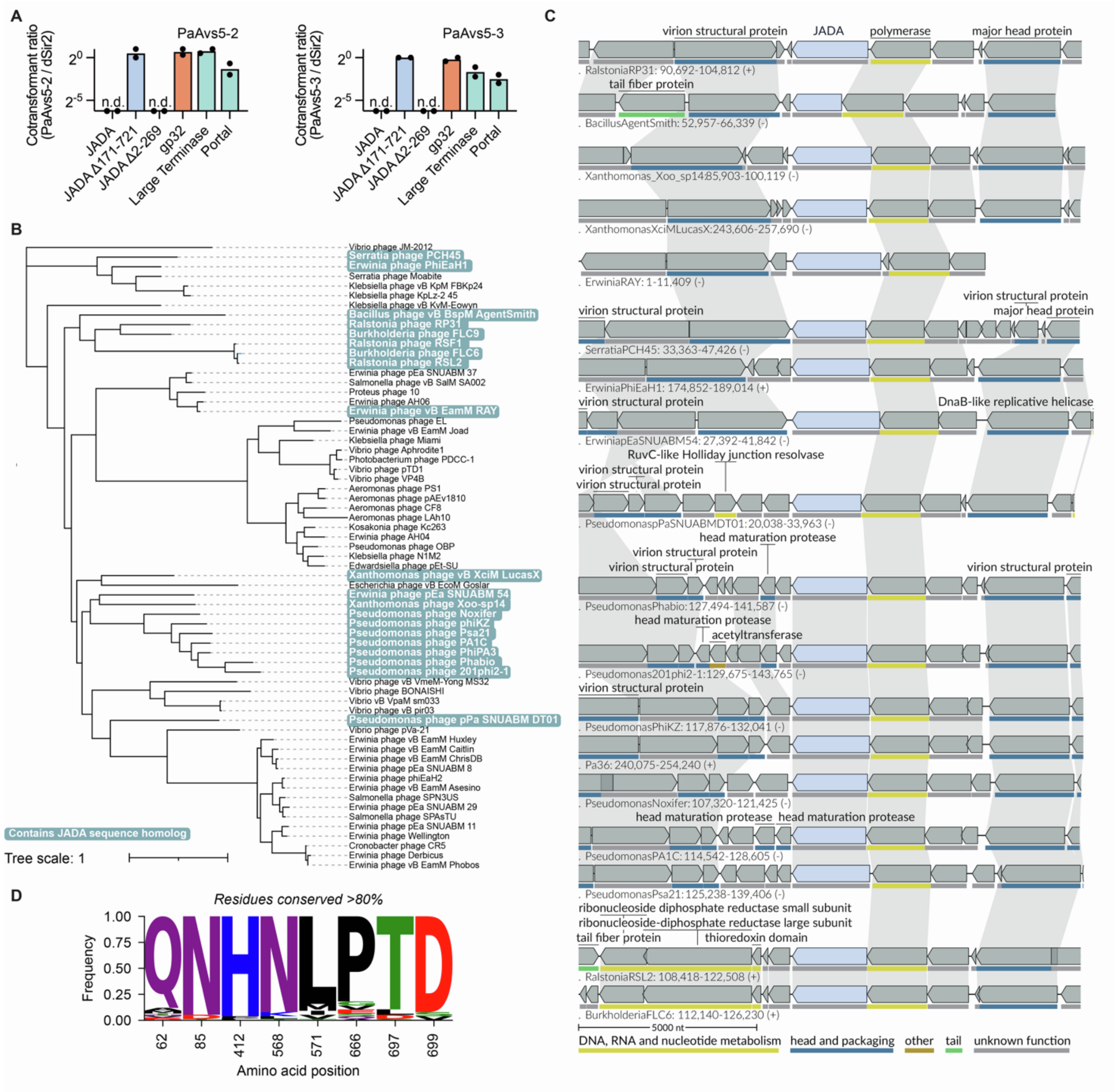

Figure S6: Conservation and Genomic Context of JADA Homologs Across Jumbo Phages, Related to Figure 6.

(A) Cotransformation assay testing activation of PaAvs5-2 and PaAvs5-3 by candidate activating genes (large terminase, portal, and JADA truncations) identified in Figure 5 of the main manuscript. Relative transformation ratios are shown for each combination, normalized to PaAvs5-1 dSir2 (N110A) control. n.d. indicates "not detectable," corresponding to zero colonies observed in transformation assays.

(B) Phylogenetic tree of nucleus-forming jumbo phages, adapted from Prichard et al. (2023) Phages encoding JADA sequence homologs are highlighted in blue.

(C) Genomic neighbourhoods of JADA homologs across representative jumbo phages. An invariant feature across all contexts is the presence of a non-virion RNA polymerase gene located immediately upstream of the JADA homolog, suggesting a conserved functional or regulatory linkage.

(D) Sequence logo showing residues conserved in >80% of JADA homologs. Residue numbering corresponds to the reference JADA protein in Pa36.

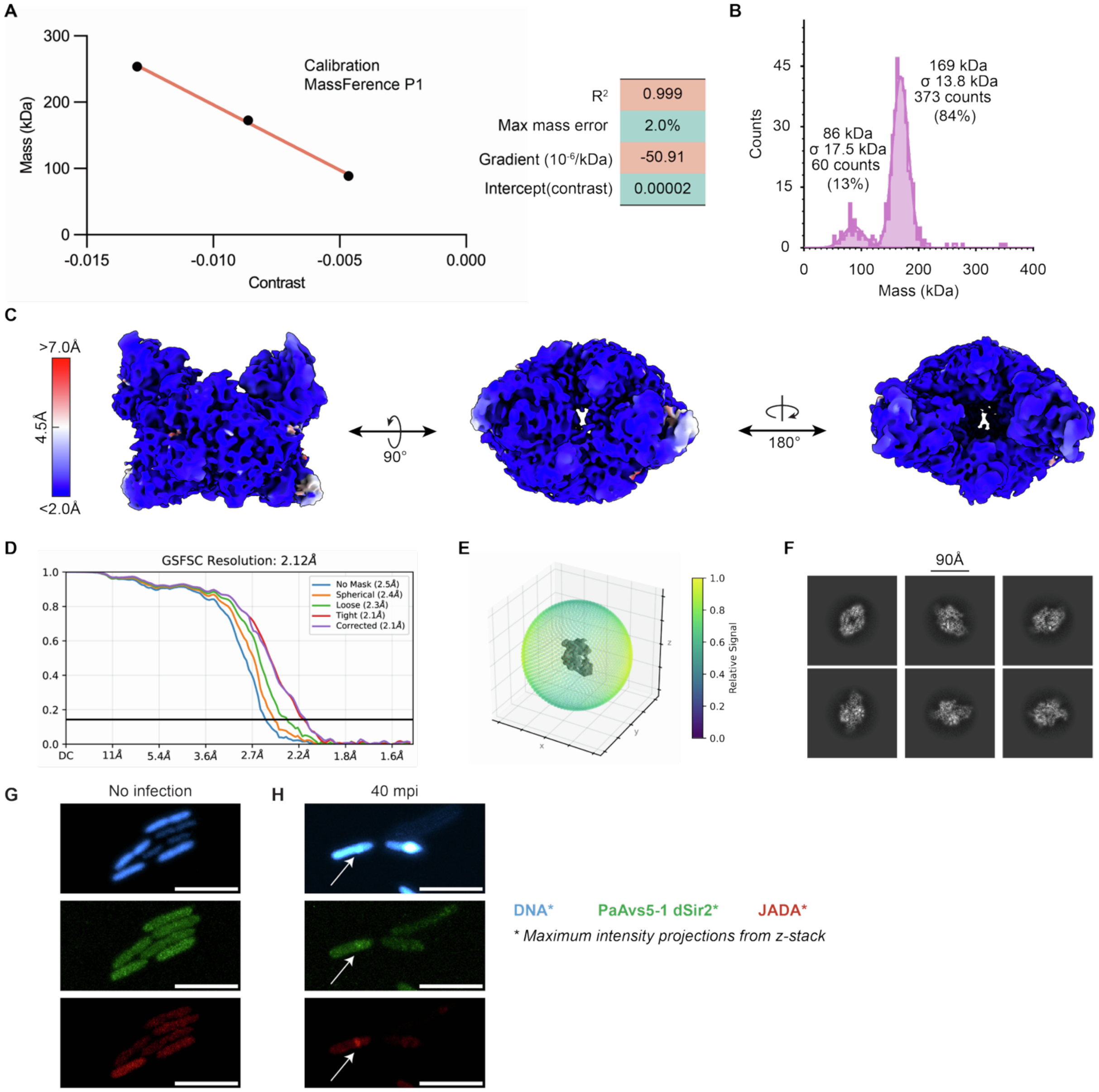

Figure S7: Cryo-EM Map Quality and Resolution Estimation of JADA Homodimer and Fluorescence microscopy of JADA, Related to Figure 6.

(A) Calibration curve generated using the MassFerence P1 internal protein standards (86, 172, and 256 kDa). The calibration yielded a linear fit with R^2 = 0.999 and a maximum mass error of 2.0%.

(B) Mass photometry of purified JADA showing two major species corresponding to the monomer (~86 kDa) and a predominant dimer (~169 kDa, 84% of total counts), confirming that JADA forms a stable homodimer in solution.

(C) Local resolution estimation of the JADA cryo-EM map, displayed with color gradient from high (blue) to low (red) resolution. Three orthogonal views of the density map are shown, indicating an overall well-resolved structure.

(D) Gold-standard Fourier shell correlation (GSFSC) curves for different masking conditions, yielding an overall resolution of 2.12 Å at the 0.143 threshold.

(E) 3D directional Fourier shell correlation (dFSC) plot shows isotropic resolution across the map.

(F) Representative 2D class averages of JADA particles, highlighting structural features and particle homogeneity.

(G)-(H) Confocal fluorescence microscopy of *P. aeruginosa* PAO1 cells co-expressing PaAvs5-1-dSir2(N110A)–mNeonGreen and JADA–mScarlet3 before infection (G) and 40 minutes post-infection (H) with phage Pa36 (MOI = 5). Prior to infection, JADA is diffuse throughout the cytosol. Following infection, JADA forms distinct foci that colocalize with DAPI-stained DNA and PaAvs5-1 foci, consistent with association at the phage nucleus. Arrows indicate representative foci. Scale bars, 5 μm.
